# A small molecule produced by *Lactobacillus* species blocks *Candida albicans* filamentation by inhibiting a DYRK1-family kinase

**DOI:** 10.1038/s41467-021-26390-w

**Published:** 2021-10-22

**Authors:** Jessie MacAlpine, Martin Daniel-Ivad, Zhongle Liu, Junko Yano, Nicole M. Revie, Robert T. Todd, Peter J. Stogios, Hiram Sanchez, Teresa R. O’Meara, Thomas A. Tompkins, Alexei Savchenko, Anna Selmecki, Amanda O. Veri, David R. Andes, Paul L. Fidel, Nicole Robbins, Justin Nodwell, Luke Whitesell, Leah E. Cowen

**Affiliations:** 1 Department of Molecular Genetics, University of Toronto, Toronto, ON Canada; 2 Department of Biochemistry, University of Toronto, Toronto, ON Canada; 3 Center of Excellence in Oral and Craniofacial Biology, Louisiana State University Health Sciences Center School of Dentistry, New Orleans, LA USA; 4 Department of Microbiology and Immunology, University of Minnesota Medical School, Minneapolis, MN USA; 5 BioZone, Department of Chemical Engineering and Applied Chemistry, University of Toronto, Toronto, ON Canada; 6 Center for Structural Genomics of Infectious Diseases (CSGID), Chicago, IL USA; 7 Department of Medicine, University of Wisconsin School of Medicine and Public Health, Madison, WI USA; 8 Department of Medical Microbiology and Immunology, University of Wisconsin, Madison, WI USA; 9 Department of Microbiology and Immunology, University of Michigan Medical School, Ann Arbor, MI 48109 USA; 10 Rosell Institute for Microbiome and Probiotics, 6100 Avenue Royalmount, Montreal, QC Canada; 11 Department of Microbiology, Immunology and Infectious Diseases, University of Calgary, Calgary, AB Canada

**Keywords:** Fungal genetics, Bacteria, Fungal biology, Microbial communities

## Abstract

The fungus *Candida albicans* is an opportunistic pathogen that can exploit imbalances in microbiome composition to invade its human host, causing pathologies ranging from vaginal candidiasis to fungal sepsis. Bacteria of the genus *Lactobacillus* are colonizers of human mucosa and can produce compounds with bioactivity against *C. albicans*. Here, we show that some *Lactobacillus* species produce a small molecule under laboratory conditions that blocks the *C. albicans* yeast-to-filament transition, an important virulence trait. It remains unexplored whether the compound is produced in the context of the human host. Bioassay-guided fractionation of *Lactobacillus*-conditioned medium linked this activity to 1-acetyl-β-carboline (1-ABC). We use genetic approaches to show that filamentation inhibition by 1-ABC requires Yak1, a DYRK1-family kinase. Additional biochemical characterization of structurally related 1-ethoxycarbonyl-β-carboline confirms that it inhibits Yak1 and blocks *C. albicans* biofilm formation. Thus, our findings reveal *Lactobacillus*-produced 1-ABC can prevent the yeast-to-filament transition in *C. albicans* through inhibition of Yak1.

## Abstract (web summary)

Alterations of the mucosal microbiota, including *Lactobacillus* bacteria, are associated with infections caused by the fungus *Candida albicans*. Here, MacAlpine et al. show that some *Lactobacillus* strains produce a small molecule that blocks *C. albicans* filamentation and biofilm formation, and thus virulence, through inhibition of a fungal kinase.

## Introduction

Fungal pathogens have an astounding, but greatly under-appreciated impact on human health worldwide, causing over a billion infections and ~1.5 million deaths annually^1^. The opportunistic pathogen *Candida albicans* is a common constituent of human mucosal microbiota and has evolved complex interactions with other members of the microbiota. As an opportunist, *C. albicans* can exploit imbalances in a host’s microbial flora, leading to diverse pathologies, including vaginal candidiasis, which occurs in ~75% of at-risk individuals at least once in their lifetime^2^. In more severe scenarios, *C. albicans* can cause life-threatening invasive infections with mortality rates of ~40%, even with current treatments^3^. This poor prognosis is in part attributable to the limited repertoire of antifungals, a challenge compounded by the frequent and widespread emergence of antifungal resistance^4^. Therefore, current research has focused on identification of strategies for treating *C. albicans* infection, including the potential of targeting virulence traits^4^.

Understanding the environmental factors that influence the switch between commensal and pathogenic states is crucial for the development of anti-virulence therapies. One attribute critical for *C. albicans* pathogenesis is its ability to switch between yeast and filamentous morphologies^5,6^. This morphological transition is important for virulence as the vast majority of mutants locked in either state are avirulent in mouse models of either systemic or superficial *C. albicans* infection^7,8^. Transition to hyphal morphology by *C. albicans* is important for virulence as it is associated with secretion of aspartic proteases and the candidalysin toxin, as well as expression of cell surface adhesins that promote tissue adhesion and invasion^5^. The transition from yeast to filaments can be induced by diverse environmental cues including exposure to serum, nutrient limitation, elevated carbon dioxide levels, as well as interactions with members of the microbiota^6^. Morphogenesis is also critical for the development of biofilms, microbial communities that can colonize indwelling devices such as catheters and display extreme resistance to xenobiotics, making them very difficult to eradicate in healthcare settings^9^.

Recent decades have witnessed enormous progress in defining the human microbiota and its profound impact on both normal physiology and disease^10^. An environment where interactions between bacteria and fungi are particularly crucial for homeostasis in healthy humans is the vaginal mucosa. This is evidenced by the increased risk of vulvovaginal candidiasis in individuals with reduced colonization by key *Lactobacillus* spp.^11,12^. Imbalances can arise from physiological changes that accompany pregnancy, the use of oral contraceptives or antibiotics, as well as poorly controlled diabetes and innate or acquired immune dysfunction. However, the mechanisms by which *Lactobacillus* spp. prevent *C. albicans* overgrowth and superficial mucosal invasion remain poorly defined. Originally it was postulated that lactic acid secreted by *Lactobacilli* reduces the mucosal pH to a level that mitigates microbial overgrowth^11^. More recently, in vitro experiments found that *Lactobacillus* spp. secrete other small molecules with bioactivity against *C. albicans*^13–16^, including metabolites that either kill the fungus^13,17^, or block the yeast-to-filament transition^14–16^. However, the identities of these secreted compounds have yet to be determined and the mechanisms by which they inhibit filamentous growth remain enigmatic.

Here, we report a *Lactobacillus*-secreted molecule that is sufficient to repress *C. albicans* morphogenesis in response to diverse filament-inducing cues in culture without impacting viability and growth of the fungus in yeast form. Using a bioassay-guided fractionation approach followed by structural elucidation, we successfully linked this activity to a defined molecular entity, 1-acetyl-β-carboline (1-ABC). Using genetic approaches, we identified the target of 1-ABC responsible for inhibition of *C. albicans* filamentation as the DYRK (dual-specificity tyrosine phosphorylation-regulated kinase)-family member, Yak1. In follow up, using a genetically-engineered system we selected for mutants with restored capacity to filament in the presence of 1-ABC. Through whole-genome sequencing, we identified single amino acid substitutions in the putative phosphatase Oca6 and in the transcription factor Rob1, each of which were shown to be necessary and sufficient to bypass the filamentation-repressive effects of the molecule. Finally, we found that in blocking the induction of filamentation, the highly structurally related compound, 1-ethoxycarbonyl-β-carboline (1-ECBC), inhibited Yak1 activity and blocked *C. albicans* biofilm formation in several co-culture models and a rat catheter infection model. Overall, this work identifies a natural product secreted by *Lactobacillus* under standard laboratory culture conditions that inhibits the yeast-to-hyphae transition and biofilm formation in *C. albicans*.

## Results

### *Lactobacillus* spp. secrete a factor that blocks *C. albicans* filamentation

**Fig. 1:**
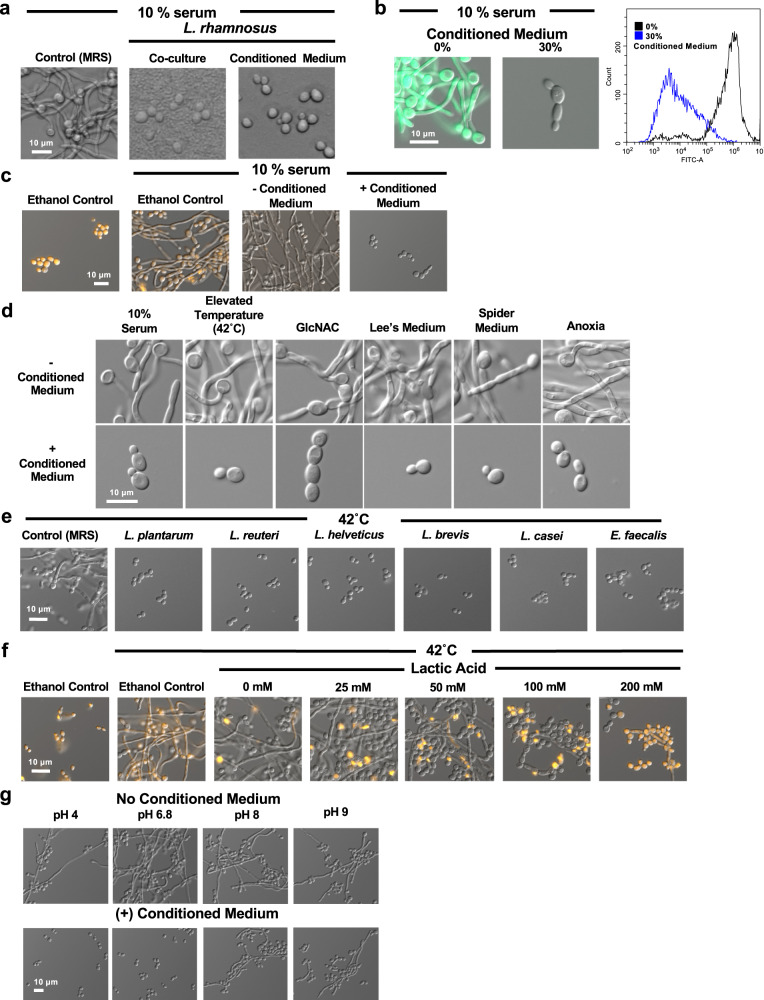
*L. rhamnosus* secretes a small molecule that inhibits *C. albicans* hyphal morphogenesis. **a** *C. albicans* grown in 10% serum at 37 °C was treated with 30% v/v MRS medium, 30% v/v MRS with *L. rhamnosus*, or 30% v/v *L. rhamnosus-*conditioned medium for 3 h. Representative images are shown of three biological replicates. **b** A *C. albicans pHWP1-GFP* reporter strain was treated with *L. rhamnosus*-conditioned medium in to 10% serum. Cells were analyzed by flow cytometry. Histogram depicts the distribution of gated events for the indicated relative fluorescence intensities. Data represent two biological replicates. Source data are provided as a Source Data file. **c** *C. albicans* was cultured in the absence or presence of 10% serum and conditioned medium, as indicated. Aliquots of yeast and serum-induced cells were treated with 70% ethanol as a control for cell death. Cells were stained with propidium iodide and fluorescence was visualized. Data represent two biological replicates. **d** *L. rhamnosus-*conditioned medium blocks *C. albicans* filamentation in response to diverse inducing cues, including elevated temperature (42 °C, 6 h, YPD), 10% serum (37 °C, 3 h, YPD), 5 mM N-acetylglucosamine (GlcNAc; 37 °C, 3 h, YPD), Lee’s Medium (37 °C, 6 h), Spider Medium (37 °C, 6 h), and growth under anerobic conditions (37 °C, 4 h, YPD). Data represent three biological replicates. **e** Conditioned-medium (30% v/v) from strains capable of blocking hyphal morphogenesis in response to elevated temperature (42 °C, 6 h) without significantly impacting growth are shown. Data represent two biological replicates. **f** Cultures of *C. albicans* were grown at 30 °C or 42 °C and subjected to a gradient of lactic acid concentrations as indicated. Cultures were treated as described in (**c**) and data represent two biological replicates. **g** *C. albicans* was incubated at 42 °C for 6 h in the absence or presence of 30% v/v *L. rhamnosus-*conditioned medium. The pH of the medium was adjusted to desired values as indicated. Data represent two biological replicates.

To investigate the mechanisms by which *Lactobacillus* affects *C. albicans* hyphal morphogenesis, we first grew the fungus in standard YPD medium supplemented with MRS bacterial culture medium (30% v/v) to which serum (10% v/v) was added as a filamentation-inducing cue. Under these conditions, *C. albicans* underwent robust filamentous growth. However, the addition of live *Lacticaseibacillus rhamnosus* (previously known as *Lactobacillus rhamnosus*^18^) under the same culture conditions blocked the yeast-to-filament transition (Fig. 1a). Next, we investigated whether live *L. rhamnosus* was required, or if the block in filamentation could be attributed to a soluble factor produced by the organism. To do so, *L. rhamnosus* was grown in MRS medium for 18 h before it was removed by filtration to yield conditioned medium. Interestingly, conditioned medium alone (30% v/v) was sufficient to block filamentation (Fig. 1a). This qualitative observation was quantified by examining effects on a strain of *C. albicans* in which the promoter of the filament-specific gene *HWP1* was placed upstream of a gene encoding green fluorescent protein (*pHWP1-GFP*). When cultures of this strain were grown at 37 °C in YPD/MRS medium containing 10% serum, robust filamentation was observed, which was accompanied by induction of bright fluorescent signal. In contrast, treatment with *Lactobacillus*-conditioned MRS medium blocked both filamentation and expression of GFP, as quantified by flow cytometry (Fig. 1b and Supplementary Fig. 1a). To determine whether the block in filamentation was due to compromised cell viability, *C. albicans* cells grown in *Lactobacillus*-conditioned medium were incubated with propidium iodide, a red-fluorescent DNA-binding dye only permeable to dead cells. *Lactobacillus*-conditioned medium blocked serum-induced filamentation without affecting *C. albicans* viability (Fig. 1c). In addition, *C. albicans* treated with *Lactobacillus*-conditioned medium proliferated at a rate comparable to untreated controls, as determined by measuring optical density of cultures over 24 h, further highlighting that conditioned-medium did not significantly impact viability and yeast-form growth (Supplementary Fig. 1b).

Next, we investigated whether the ability of *Lactobacillus*-conditioned medium to block filamentation was restricted to serum induction, or whether impairment of hyphal morphogenesis was conserved across diverse filament-inducing cues. Supplementation with *L. rhamnosus*-conditioned medium blocked *C. albicans* hyphal morphogenesis in response to all inducing cues examined, including elevated temperature (42 °C), alternative carbon source (N-acetylglucosamine, GlcNAc), amino acid deficiency (Lee’s Medium), nutrient deficiency (Spider Medium), and anoxia (Fig. 1d). To determine if this activity was specific to *L. rhamnosus* or more broadly conserved across diverse bacterial species and genera, we tested a collection of 44 bacterial species and a collection of nine industrial probiotic bacterial strains for their ability to block *C. albicans* filamentation induced by elevated temperature. Most bacterial species tested significantly impaired *C. albicans* growth, resulting in a block in hyphal morphogenesis. However, only conditioned MRS media from multiple industrial *Lactobacillus* species, as well as isolates of *Limosilactobacillus reuteri* (previously known as *Lactobacillus reuteri*^18^) and *Enterococcus faecalis* from mice, were able to block *C. albicans* filamentation at 42 °C without significantly impacting growth (Fig. 1e and Supplementary Fig. 2). While all bacteria were cultured in standard MRS medium to promote their adequate growth, it remains unknown whether other bacterial species would produce a soluble factor capable of suppressing *C. albicans* filamentation under alternate conditions. Thus, select *Lactobacillus* spp. and *E. faecalis* appear to share the specific ability to impair *C. albicans* hyphal morphogenesis upon growth in MRS medium through production of a soluble factor(s). We focused on *Lactobacillus* spp. as previous work determined *E. faecalis* secretes a small peptide that blocks *C. albicans* hyphal morphogenesis^19^.

### *Lactobacillus*-secreted 1-ABC blocks *C. albicans* filamentation

*Lactobacillus* spp. are characterized by their ability to produce lactic acid as the major end-product of their carbon metabolism^20^. Thus, we assessed whether lactic acid was sufficient to block filamentation under conditions that did not impair fungal viability. While treatment with lactic acid (100–200 mM) blocked the yeast-to-filament transition, it was accompanied by a significant reduction in viability, as detected by propidium iodide staining (Fig. 1f). Furthermore, we found that although the anti-filamentation activity of *Lactobacillus-*conditioned medium required acidic conditions (pH < 6.8), acidic pH alone was not sufficient to block filamentation (Fig. 1g). Therefore, we reasoned lactic acid is not the soluble factor produced by *Lactobacillus* that is responsible for blocking *C. albicans* hyphal morphogenesis without impairing viability.

To characterize the nature of the soluble component responsible for bioactivity, we subjected *Lactobacillus-*conditioned medium to a panel of enzymatic and physical treatments. Consistent with the expectation that significant protease and nuclease activity would be present in *Lactobacillus*-conditioned medium, filamentation inhibitory activity was unaffected by boiling or by treatment with DNase I, RNase A, or Proteinase K (Supplementary Fig. 1c), suggesting it was not due to DNA, RNA, or protein, but rather a small molecule distinct from lactic acid.

**Fig. 2:**
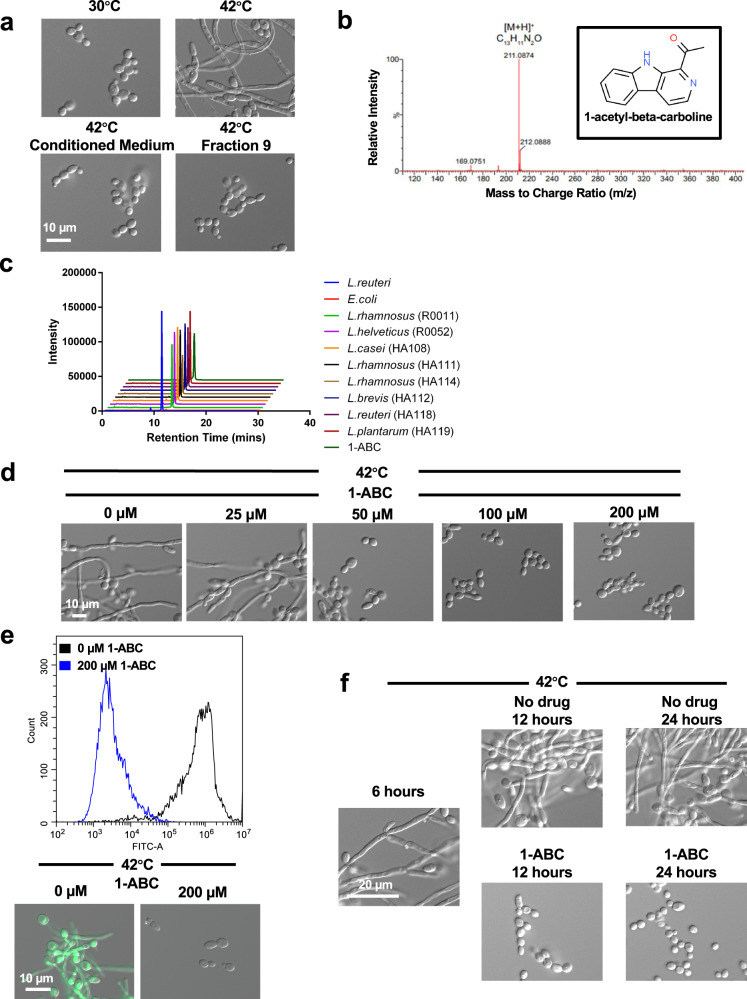
*Lactobacillus* inhibit *C. albicans* filamentation through the secretion of 1-ABC. **a** Separation of *L. reuteri-*conditioned medium by reverse-phase HPLC using a C18 column identified fraction 9 with activity comparable to the *L. reuteri-*conditioned medium in blocking *C. albicans* filamentation induced by high temperature (42 °C, 6 h). Data represent two biological replicates. **b** Mass spectrum of the single molecular entity in fraction 9 yielded a mass of 210.0796 Da consistent with the formula C_13_H_11_N_2_O (theoretical (Da): 210.0793). Structure of the molecule was determined by NMR. **c** Analysing metabolite composition of conditioned media from a variety of bacterial isolates demonstrates that 1-ABC is produced across the *Lactobacillus* genus. Shown is an extracted-ion chromatogram (211.0874 + /− 0.05 *m/z*) of 1-ABC proton adduct for each strain including the negative control *E. coli* and a 1-ABC standard. Source data are provided as a Source Data file. **d** 1-ABC inhibits *C. albicans* filamentation in a concentration-dependent manner. Cells were grown at 42 °C for 6 h in the absence or presence of a gradient of 1-ABC. Data represent three biological replicates. **e** Quantification of the inhibitory effect of 1-ABC on filamentation of a *C. albicans pHWP1-*GFP reporter strain in response to high temperature (42 °C, 6 h). Histogram is representative of two biological replicates and depicts relative fluorescence intensity (FITC-A) of events. Source data are provided as a Source Data file. **f** 1-ABC blocks *C. albicans* filamentation after the program is initiated by growth at 42 °C for 6 h. At this point, cells were left untreated (no drug), or treated with 200 μM 1-ABC and left to grow for an additional 6 h (12 h total) or an additional 18 h (24 h total). Data represent two biological replicates.

To identify the metabolite(s) responsible for this observed inhibition, we employed a bioactivity-guided purification strategy leading to identification of a single molecular entity capable of blocking *C. albicans* hyphal morphogenesis (Figs. 2a, b). The structure of this compound, 1-ABC, was readily elucidated via 1D and 2D nuclear magnetic resonance (NMR) techniques and the expected fragmentation pattern confirmed via tandem mass spectrometry (Supplementary Fig. 3a–c). Although commonly produced by plants and a few bacterial species^21,22^, β-carbolines have not previously been reported to be produced by *Lactobacillus*. Interestingly, 1-ABC was also detected in conditioned medium prepared from nine additional strains of *Lactobacillus* capable of blocking *C. albicans* hyphal morphogenesis but was not detected in the supernatant of *Escherichia coli* cultures, a species unable to block the *C. albicans* yeast-to-filament transition (Fig. 2c). It remains unexplored whether 1-ABC is produced in the context of the host.

To confirm that 1-ABC was sufficient to block *C. albicans* filamentation, a synthetic compound was purchased from a commercial source and incubated with *C. albicans* grown at 42 °C. Replicating the effect of *Lactobacillus-*conditioned medium (Fig. 1), 1-ABC blocked *C. albicans* filamentation, as visualized by microscopy (Fig. 2d) and quantified by flow cytometry using our *pHWP1-GFP* reporter strain (Fig. 2e and Supplementary Fig. 1), without affecting cell viability (Supplementary Fig. 3d). 1-ABC was able to block *C. albicans* filamentation in response to all inducing cues tested (Supplementary Fig. 3e). To further characterize its activity, synthetic 1-ABC was tested not only for the ability to impede the initiation of filamentous growth, but also the ability to block filamentation in a culture that had already initiated this developmental transition. Cultures of *C. albicans* were incubated for 6 h at 42 °C, at which point they were either solvent treated or treated with 1-ABC for an additional 6 or 18 h. Solvent-treated controls maintained robust filamentation, whereas cultures treated with 1-ABC were observed in the yeast form at both time points (Fig. 2f). Collectively, these results identify 1-ABC as a bioactive compound produced by *Lactobacillus* spp. in culture which is capable of blocking *C. albicans* hyphal morphogenesis.

### The DYRK1-family kinase Yak1 is the putative target of 1-ABC

**Fig. 3:**
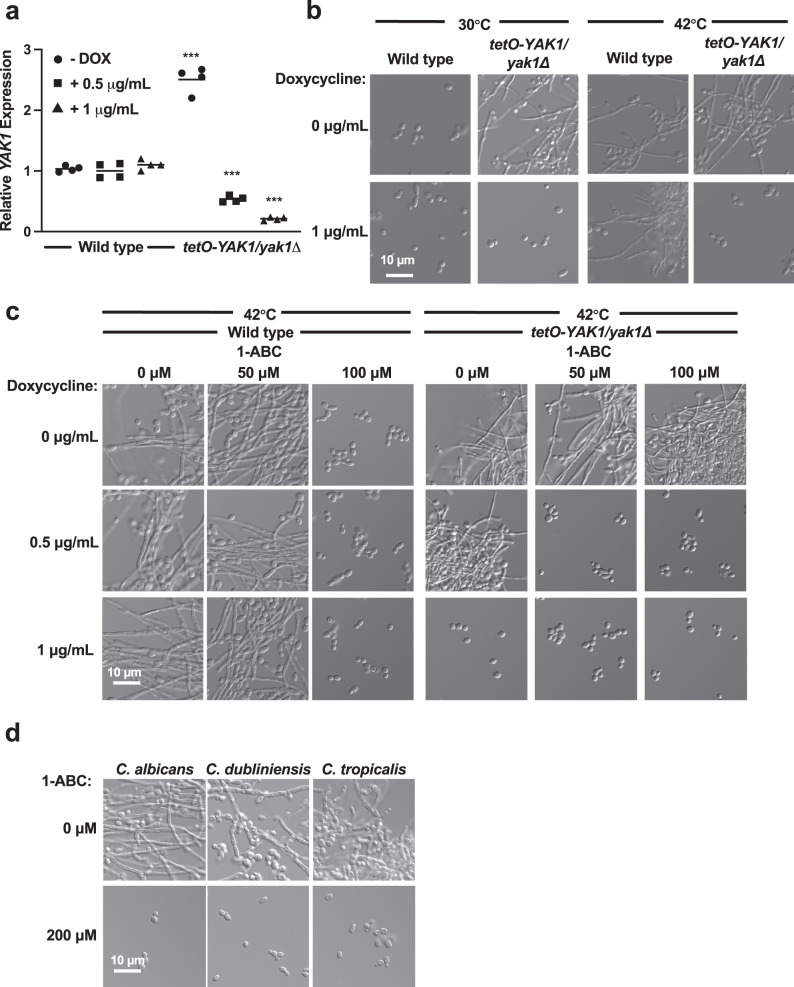
Yak1 is a putative target of 1-ABC. **a** *YAK1* transcript levels are elevated in a *tetO-YAK1/yak1Δ* strain in the absence of doxycycline (DOX) and repressed in the presence of DOX. Cells were grown at 30 °C for 3 h in the absence and presence of 0.5 µg/mL or 1 µg/mL doxycycline (DOX), as indicated. Transcript levels were normalized to *GPD1* and are relative to the wild type no DOX control. Data are presented as mean of technical quadruplicates. Significance of differences between the wild type no DOX and all treatment groups was determined by a one-way ANOVA; ***p value < 0.0001. Measure of center represents the mean of the data. Source data are provided as a Source Data file. **b** Overexpression of *YAK1* induces filamentation in the absence of inducing cues, and transcriptional repression of *YAK1* blocks filamentation in response to elevated temperature. Cells were grown under non-filament inducing conditions (30 °C, 24 h) or filament-inducing conditions (42 °C, 24 h) in the absence and presence of 1 µg/mL DOX, as indicated. Data represent three biological replicates. **c** Reduction of *YAK1* levels confers hypersensitivity to the filament-repressing effects of 1-ABC. *C. albicans* was grown at 42 °C for 6 h in the absence or presence of differing concentrations of DOX and in the absence and presence of 50 µM 1-ABC. Data represent three biological replicates. **d** 1-ABC inhibits filamentation of *C. dubliniensis* and *C. tropicalis*. Cells were grown at 42 °C for 6 h in the absence or presence of 200 µM 1-ABC. Data represent two biological replicates.

β-carbolines have been reported to cause diverse biological effects and are well-characterized inhibitors of mammalian dual-specificity tyrosine phosphorylation-regulated kinases (DYRKs)^23^. To determine whether 1-ABC inhibits Yak1, the sole DYRK-family member expressed in *C. albicans*, we took advantage of a genetically engineered strain in which one allele of the kinase is deleted and the remaining allele is placed under a tetracycline-repressible promoter (*tetO-YAK1/yak1Δ*). In the absence of the tetracycline analog doxycycline (DOX), *YAK1* was overexpressed in the *tetO-YAK1/yak1Δ* strain relative to wild type, and this overexpression was sufficient to induce filamentous growth in the absence of an inducing cue (Figs. 3a, b). In contrast, DOX (0.5–1 μg/mL) significantly repressed *YAK1* expression in the *tetO-YAK1/yak1Δ* background relative to wild type, which was sufficient to block filamentation normally induced by growth at elevated temperature (Fig. 3a, b). This finding is consistent with a previous report that Yak1 is required for filamentation^24^.

Next, we determined whether transcriptional alteration of *YAK1* expression conferred differential susceptibility to the effects of 1-ABC, which would be expected if the compound inhibits Yak1 function. To do so, wild-type and *tetO-YAK1/yak1Δ* strains were treated with both DOX and 1-ABC. It was first determined that treatment with 50 µM 1-ABC was not sufficient to block filamentation in wild-type *C. albicans* (Fig. 3c). However, when *YAK1* expression is suppressed with an intermediate concentration of DOX that does not inhibit filamentation in the *tetO-YAK1/yak1Δ* strain (0.5 µg/mL), filamentation was blocked by treatment with 50 µM 1-ABC (Fig. 3c). This result is consistent with reduced *YAK1* expression conferring hypersensitivity to 1-ABC. In addition, in the *tetO-YAK1/yak1Δ* background, overexpression of *YAK1* in the absence of DOX maintained *C. albicans* filamentation despite the presence of 100 µM 1-ABC (Fig. 3c), consistent with enhanced expression conferring resistance to the effects of the compound.

Interestingly, Yak1 is conserved in other *Candida* spp. that undergo the yeast-to-hyphae transition, including *Candida dubliniensis* and *Candida tropicalis*. Thus, we tested the ability of the β-carboline to inhibit filamentation in these non-*albicans Candida* species. Indeed, 1-ABC inhibited filamentation in both species of *Candida*, suggesting Yak1 may be necessary for the morphogenetic transition in multiple *Candida* species (Fig. 3d).

### Mutations in *OCA6* or *ROB1* block filament-repressive activity of 1-ABC

**Fig. 4:**
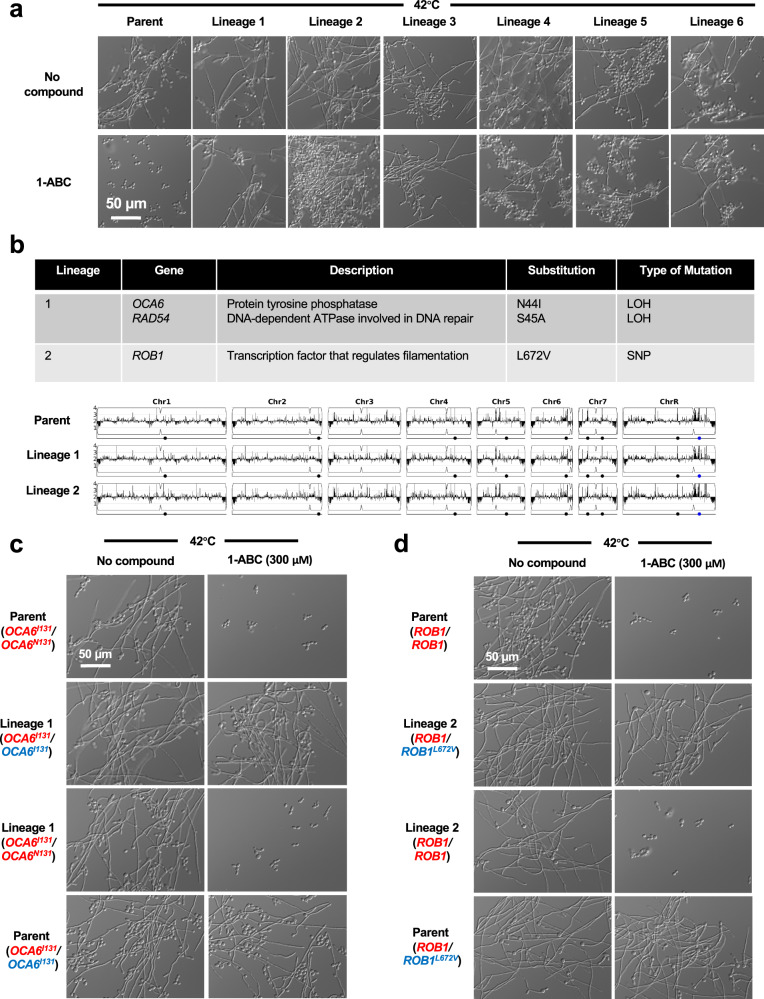
Mutations in *OCA6* and *ROB1* enable *C. albicans* filamentation despite exposure to 1-ABC. **a** A selection-based strategy resulted in the identification of six independent lineages with a restored ability of *C. albicans* to filament in the presence of 1-ABC. The filamentation phenotype was validated in YPD medium at 42 °C in the presence of 1-ABC (300 µM). Data represent two biological replicates. **b** Summary of whole-genome sequencing results for two of the evolved lineages. All identified loss-of-heterozygosity events or single nucleotide polymorphisms that occurred within open reading frames of each lineage are shown. Results were independently validated using Sanger sequencing. Whole-genome sequencing analysis of Lineages 1 and 2 did not detect any copy number variants relative to the parental strain. Copy number variant analysis was conducted and visualized using the Y-MAP software^63^. **c** The *OCA6* mutation in Lineage 1 is necessary and sufficient for the filamentation phenotype. A wild-type *OCA6* allele from the parental strain (red) was used to replace the mutant *OCA6* allele (blue) from Lineage 1. Similarly, the mutant *OCA6* allele from Lineage 1 (blue) was used to replace the wild-type *OCA6* allele (red) in the parental background. Strains were grown at 42 °C for 6 h and data represent two biological replicates. **d** The *ROB1* mutation in Lineage 2 is necessary and sufficient for the filamentation phenotype. A wild-type *ROB1* allele from the parental strain (red) was used to replace the mutant *ROB1* allele (blue) from Lineage 2. Similarly, the mutant *ROB1* allele from Lineage 2 (blue) was used to replace the wild-type *ROB1* allele (red) in the parental background. Strains were grown at 42 °C for 6 h and data represent two biological replicates.

To investigate the pathways through which Yak1 governs filamentation, we used a selection strategy to identify mutants capable of filamenting in the presence of 1-ABC. To do so, we genetically engineered a strain that harbored a dominant nourseothricin (NAT)-selectable marker expressed under the control of the filament-specific promoter, *pHWP1*. In this system, filamentation is required for survival in the presence of NAT, a situation which does not exist in the host where filamentation plays important roles, including those important for virulence^25^. Integration of this construct into the *C. albicans* genome confers NAT resistance only upon induction of a filamentation-specific gene expression program^26^. Selection experiments were performed in parallel using independent cultures of our *C. albicans* strain harboring the *pHWP1-*NAT construct. Each culture was plated on rich medium containing 1-ABC (300 µM) and NAT (200 µg/mL) and allowed to grow at 42 °C for three days. Colonies displaying NAT resistance were re-streaked on selection plates to establish six independent lineages, and the filamentation phenotype was verified (Fig. 4a). Notably, these lineages did not display constitutive filamentation in the absence of an inducing cue (Supplementary Fig. 4a). To determine the genetic basis for the restoration of filamentation in the presence of 1-ABC, we performed whole-genome sequencing on a representative colony from each of the six independent lineages. No significant aneuploidies were observed (Fig. 4b and Supplementary Fig. 4b), however, mutations encoding non-synonymous substitutions or loss-of-heterozygosity within open reading frames were detected and validated using Sanger sequencing (Fig. 4b and Supplementary Fig. 4b). The mutations included loss-of-heterozygosity within *OCA6*, a gene encoding a putative phosphatase^27^, and a single nucleotide polymorphism in *ROB1*, encoding a transcription factor with important roles in *C. albicans* morphogenesis^28^. Mutations were also identified in the other four evolved lineages, which will require further investigation to determine their impact on *C. albicans* resistance to 1-ABC.

To determine if the mutations identified in *OCA6* and *ROB1* were necessary and sufficient to confer resistance to the effects of 1-ABC, an allele-swap strategy was utilized. Mutant alleles of either *OCA6* or *ROB1* from the respective evolved lineages were replaced with a wild-type allele. For both genes, the swap restored sensitivity to the filamentation inhibitory activity of 1-ABC, confirming that these mutations were necessary for 1-ABC resistance of the lineages in which they were detected (Fig. 4c, d). To determine if the mutations were sufficient for 1-ABC resistance, one of the wild-type alleles in the parental strain was replaced with the mutant allele of either *OCA6* or *ROB1* identified in the selection experiments. For both replacements, the mutation was sufficient to confer resistance to 1-ABC as the allele-swapped parental strains were able to filament in the presence of the compound (Figs. 4c, d). Both the *OCA6* loss-of-heterozygosity and the *ROB1* single nucleotide polymorphism were predicted to be gain-of-function mutations, as homozygous deletion of *OCA6* (Supplementary Fig. 4c) or heterozygous deletion of *ROB1* (Supplementary Fig. 4d) did not alter the *C. albicans* response to 1-ABC in a wild-type genetic background.

**Fig. 5:**
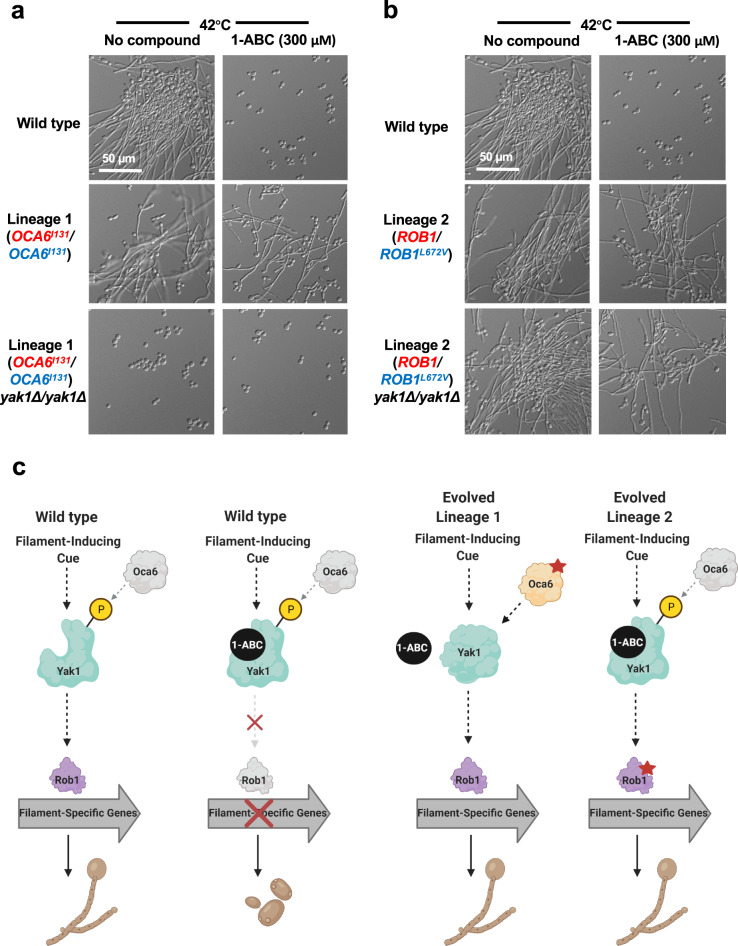
Oca6 acts upstream and Rob1 acts downstream of Yak1 to enable *C. albicans* filamentation. **a** Deletion of *YAK1* in Lineage 1 blocked *C. albicans* filamentation in response to high temperature. Strains of *C. albicans* were grown in YPD at 42 °C for 6 h in the absence or presence 1-ABC, as indicated. Data represent two biological replicates. **b** Deletion of *YAK1* in Lineage 2 did not affect the mutant phenotype and *C. albicans* continued to filament in the presence of 1-ABC. Strains of *C. albicans* were grown as described in (**a**). Data represent two biological replicates. **c** Model depicting proposed mechanisms by which Yak1, Oca6, and Rob1 interact in regulating *C. albicans* hyphal morphogenesis in the absence or presence of 1-ABC. Created with BioRender.com.

Next, we investigated the epistatic relationship between *OCA6* or *ROB1* and *YAK1* with respect to the ability to regulate *C. albicans* morphogenesis. To do so, we generated a *yak1* homozygous deletion mutant in each of the two evolved lineages. Deletion of *YAK1* resulted in a block in filamentation in the lineage carrying the Oca6 substitution (N44I, Lineage 1), which we had found to confer 1-ABC resistance (Fig. 5a). This finding suggests that Oca6 functions upstream of Yak1 to govern *C. albicans* filamentation. In light of this relationship, mutational dysregulation of Oca6 phosphatase activity would be expected to impair the ability of 1-ABC to inhibit Yak1, either directly through dephosphorylation of autoregulatory residues within the kinase itself or indirectly through effects on other substrates^29^ (Fig. 5c). In contrast to Lineage 1, deletion of *YAK1* did not affect the ability of Lineage 2 (harboring a Rob1 L672V substitution) to filament in response to elevated temperature or its resistance to 1-ABC (Fig. 5b), placing the transcription factor downstream of Yak1 in regard to regulation of morphogenesis (Fig. 5c).

### β-carbolines inhibit key virulence traits in *C. albicans*

As a prelude to virulence studies, we assessed the stability of 1-ABC’s bioactivity by incubating the compound with human liver-derived HepG2 cells. The compound was incubated overnight with these metabolically active cells in serum-supplemented medium at 37 °C. We then measured the ability of the supernatant medium to block *C. albicans* filamentation in response to the inducing cue of 5% serum at 37 °C. Unfortunately, a loss of bioactivity was observed (Supplementary Fig. 5a, b). Serum-supplemented tissue culture medium alone did not inactivate 1-ABC, leading us to conclude that cellular metabolism rather than serum instability was most likely responsible for inactivation (Supplementary Fig. 5b). To overcome this liability, we investigated the stability of a small series of commercially available structural analogs using the same experimental approach. All β-carboline scaffolds tested blocked *C. albicans* filamentation with varying degrees of potency (Supplementary Fig. 5a). Unfortunately, most compounds were still susceptible to degradation by HepG2 cells in culture (Supplementary Fig. 5a). Fortunately, a hydrochloride salt of 1-ethoxycarbonyl-beta-carboline (1-ECBC), was found to block *C. albicans* filamentation and to remain active after incubation with HepG2 cells (Supplementary Fig. 5b).

**Fig. 6:**
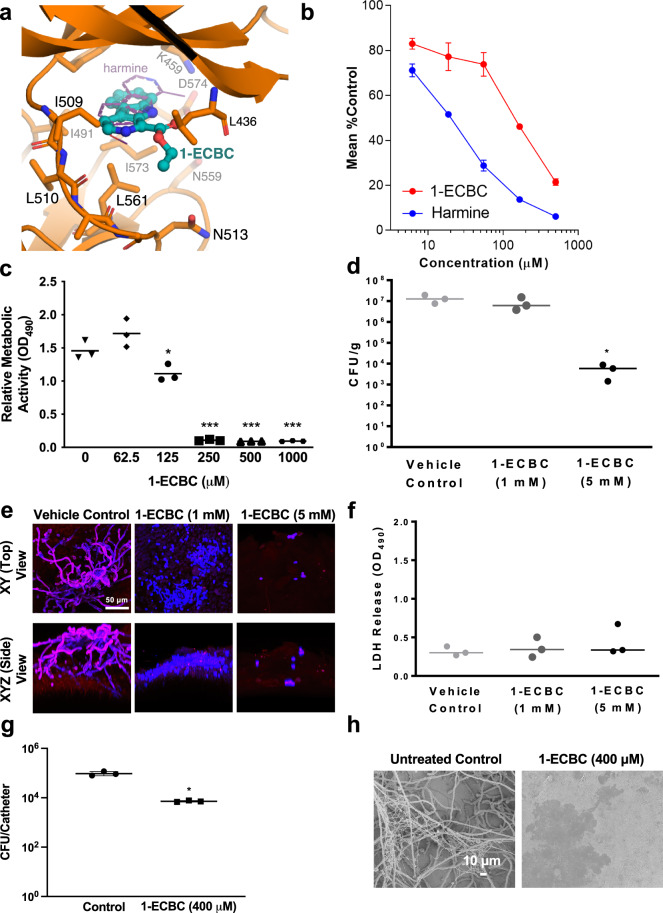
1-ECBC inhibits virulence of *C. albicans*. **a** Homology model of 1-ECBC bound to the Yak1 kinase domain. Critical residues important for the interaction are depicted. For comparison, harmine bound to mammalian DYRK1A (PDB 3ANR^70^) is shown as a thin purple line. **b** 1-ECBC and harmine inhibit Yak1 activity in a concentration-dependent manner. Error bars represent ±SEM of five technical replicates. Data are representative of two biological replicates. **c** 1-ECBC prevents the development of *C. albicans* biofilms in vitro. Biofilms were grown for 24 h in the absence or presence of 1-ECBC as indicated. The XTT dye was used to evaluate metabolic activity. Data is an average of five technical replicates and representative of two biological replicates. Measure of center represents the mean of the data. Significance of differences between wild type and all treatment groups was determined by one-way ANOVA; *p value < 0.0136, ***p value < 0.0001. **d** 1-ECBC reduces tissue fungal burden on a murine vaginal explant (*n* = 3). Significance of differences between the vehicle control and all compound treatment groups was determined by one-way ANOVA; *p value = 0.0284. Measure of center represents the mean of the data. **e** Fluorescence microscopy of *C. albicans* biofilms on vaginal explants treated with 1-ECBC. Blue indicates calcofluor white staining and red indicates concanavalin red staining. All images at ×600 magnification (scale bar is 50 μm). Data represent two biological replicates. **f** 1-ECBC prevents biofilm formation without causing tissue damage in vaginal explants (*n* = 3). LDH release of vaginal tissue following inoculation with wild-type *C. albicans* and treatment with or without 1-ECBC for 24 h. Measure of center represents the mean of the data. **g** 1-ECBC (400 μM) prevents *C. albicans* biofilm formation in a rat catheter model^35^. Serial dilutions of catheter fluid were plated for viable fungal colony counts in triplicate. Data are presented as mean colony forming units (CFU) per catheter for three technical replicates: one female rat catheter per treatment (*P = 0.015, paired two-sided *t* test). Measure of center represents the mean of the data. **h** Scanning electron microscopy (SEM) images of biofilms formed on catheters. Images are representative of catheters from three biological replicates. All source data for this figure are provided as a Source Data file.

To confirm that 1-ECBC inhibits filamentation by targeting Yak1, as proposed for 1-ABC, we first constructed a homology model for the structure of *C. albicans* Yak1 kinase domain based on previously published crystallographic data for DYRK-family kinases. Using this model, we observed a good fit upon docking 1-ECBC into the Yak1 ATP-binding pocket (predicted binding energy −8.5 kcal/mol) with a binding mode that is highly conserved with experimental data for co-crystal structures of the β-carboline harmine in complex with mammalian DYRK1A (Fig. 6a). To obtain biochemical evidence that 1-ECBC inhibits Yak1 kinase activity, we generated a strain of *C. albicans* with both alleles of *YAK1* modified to overexpress a protein C-terminally tagged with a 6X His-3X Flag epitope. The kinase was purified via affinity precipitation using anti-Flag beads and eluted with 3X-Flag peptide. A luciferase-based ADP-Glo kit (Promega) was used to detect ADP production upon incubation of affinity purified Yak1 with the canonical substrate DYRKtide^30^. As expected, 1-ECBC and harmine, a known β-carboline scaffold DYRK inhibitor, exhibited concentration-dependent inhibition of Yak1 activity (Fig. 6b).

Having confirmed 1-ECBC as an inhibitor of Yak1, we moved forward to investigate a potential therapeutic application for the compound by assessing its effects on *C. albicans* biofilm formation. Previous work has shown that both *Lactobacillus* and *Lactobacillus*-conditioned medium can inhibit biofilm formation in vitro^14,31,32^. To begin, we measured the ability of 1-ECBC to block biofilm formation in vitro^33^. Wild type *C. albicans* was allowed to adhere to serum-coated plastic for 90 min, wells were subsequently washed, and re-fed with medium supplemented with varying concentrations of 1-ECBC. After 24 h, wells were washed and the formazan dye XTT added to quantify metabolic activity. 1-ECBC at concentrations of ≥125 μM significantly reduced *C. albicans* biofilm formation relative to the vehicle control (Fig. 6c).

Next, we evaluated the ability of 1-ECBC to block *C. albicans* biofilm formation in more complex models. The first model was based on vaginal explants from mice^34^. Briefly, vaginal tissue was resected, and portions mounted on pedestals in 24-well plate format with the mucosal surface facing upwards. The mucosa was overlaid with wild-type *C. albicans* in the absence or presence of buffer containing 1-ECBC. Following 24-h incubation, the tissue mounts were gently rinsed. Half the tissue was homogenized and plated to assess colony forming units (CFUs), and the other half was fixed, stained, and imaged by confocal microscopy to assess biofilm architecture. 1-ECBC (5 mM) was able to block vaginal biofilm formation and reduce *C. albicans* CFUs without causing damage to the underlying mucosa as monitored by the sensitive indicator of LDH release (Fig. 6d, e, and f). Although 1 mM treatment with 1-ECBC did not significantly reduce fungal CFU, it did prevent maturation of the biofilm architecture (Fig. 6e). Finally, we assessed the ability of 1-ECBC to block biofilm formation in a standard model of *C. albicans* catheter colonization^35^. Silastic central venous catheters implanted in rats were infused with a fungal inoculum (1 × 10^6^ cells/mL) prepared in saline with or without addition of 1-ECBC (400 μM). Biofilm formation was allowed to proceed over a 24-h period. Catheters were then removed and fungal burden adherent to the catheter assessed by CFU assay. To assess effects on biofilm morphology, catheters were processed and imaged by scanning electron microscopy (SEM). In this model, 1-ECBC reduced the recovered CFU by more than 10-fold compared to the untreated control (Fig. 6g) and mature biofilm development was virtually eradicated (Fig. 6h). It remains unclear whether these results are due to a reduction in biofilm growth, reduced *C. albicans* cell adhesion, or both. These experiments provide evidence that with further development, β-carbolines could provide an effective approach to prevent biofilm formation on mucosal surfaces and implanted medical devices.

## Discussion

As the frequency of antifungal resistance continues to rise, targeting virulence traits provides an exciting strategy for improving outcomes in patients with mucosal and systemic fungal infections. Here, we report that 1-ABC, a natural product produced by *Lactobacillus* spp. under standard culture conditions can inhibit a key virulence trait in *C. albicans*, the yeast-to-filament transition. As a component of the *Lactobacillus* secretome, 1-ABC was highly effective in blocking filamentation of *C. albican*s in response to a wide variety of host-relevant inducing stimuli in culture. We also identified Yak1 as the molecular target responsible for the anti-filamentation activity of 1-ABC and other closely related β-carbolines. Further mechanistic insights were gained through identification of mutations in genes encoding the putative phosphatase Oca6 and the transcription factor Rob1, each of which conferred resistance to the filamentation-repressive effects of 1-ABC. We also showed treatment with the β-carboline 1-ECBC was effective in blocking biofilm formation using in vitro and in vivo models. Overall, this work provides molecular insights into how 1-ABC, a β-carboline found to be produced by *Lactobacillus* spp. under standard culture conditions, acts to repress the *C. albicans* yeast-to-filament transition by inhibiting Yak1.

Our discovery of *Lactobacillus*-secreted 1-ABC highlights a rich, relatively underexplored resource of biologically active compounds. Human commensal bacteria were not previously known to produce 1-ABC, and fascinating questions remain as to how and why they produce compounds of this chemical class under standard culture conditions, including growth in MRS medium. Notably, it is possible that other bacterial species produce 1-ABC or other beta-carbolines if grown under different culture conditions given that secondary metabolite production is heavily influenced by environmental conditions. Future experiments are also needed to determine if β-carbolines are produced under natural conditions, including the female vaginal tract. β-carbolines are best characterized as secondary metabolites of plants^21^, although a few species of deep-sea marine bacteria are also reported to produce them^22^. Given our observation that multiple β-carbolines inhibit *C. albicans* filamentous growth, determining whether other structurally-related β-carbolines are produced by *Lactobacillus* spp. could reveal additional molecules with greater therapeutic potential. Our findings expand the repertoire of molecules secreted by bacteria that modulate *C. albicans* hyphal morphogenesis^34,36–42^. In the lungs of patients with cystic fibrosis, quorum-sensing molecules produced by *Pseudomonas aeruginosa* and *Burkholderia* spp. mediate important inter-kingdom interactions. These compounds exert a selective pressure to repress the *C*. *albicans* filamentation program^43^. In contrast, commensal bacteria are also implicated in enabling *C. albicans* to maintain the capacity to undergo morphogenesis in the mammalian gut. Serial passage of *C. albicans* through the gastrointestinal tracts of antibiotic-treated mice led to rapid generation of low-virulence strains unable to form hyphae, while only virulent filamentation-competent *C. albicans* strains persisted when an intact microbiota was present^44^. Fungi also secrete factors that regulate bacterial pathogenesis. As an example, *Saccharomyces boulardii* can produce proteases or phosphatases that inactivate toxins produced by intestinal bacteria such as *Clostridium difficile* and *E. coli*^45,46^. Overall, continued exploration of the interactions between commensal organisms in vitro can advance our understanding of how bacteria can influence fungal biology.

Coupling genetic analyses with computational modeling and biochemical approaches, we found that β-carbolines block *C. albicans* hyphal morphogenesis by inhibiting the DYRK-family member Yak1. The signaling pathways through which Yak1 regulates *C. albicans* morphogenesis remain largely unknown. In *S. cerevisiae*, Yak1 acts downstream of Ras1 in the PKA pathway^47^, a core signal transduction cascade that orchestrates filamentation in response to diverse inducing cues. While the PKA phosphorylation sites described in *S. cerevisiae* Yak1 are conserved in *C. albicans*, other studies suggest that *S. cerevisiae* Yak1 function may also be regulated in a PKA-independent manner^48–50^. To explore Yak1 signaling in *C. albicans*, we used a genetic selection system coupled with genome sequencing to identify mutations capable of restoring filamentation despite the presence of 1-ABC. This approach identified functional connections, implicating Oca6 upstream of Yak1 and Rob1 downstream of Yak1. Additional mechanistic insights may be revealed through further analysis of four additional resistant lineages in which we identified individual loss of heterozygosity events. There may be additional functional connections with Yak1 or alternate pathways that can bypass the block in filamentation induced by β-carbolines, given the complexity of cellular signaling governing *C. albicans* hyphal morphogenesis.

Protein kinases remain the most extensively studied target class for drug discovery efforts across diverse disease indications, but they are just beginning to be considered in the development of antifungals. Encouragingly, several structurally diverse kinases, including Protein kinase C (Pkc1), Target of rapamycin kinase (Tor1), and the casein kinase I family-member Yck2 show promising therapeutic potential^51–54^. Expanding this repertoire, we highlight the potential of the DYRK-family member Yak1 as an antifungal target. Although previous work reported that *yak1*-deletion mutants did not exhibit virulence defects in mouse models of systemic and oral candidiasis^24^, targeting Yak1 could well prove effective in the prevention of *C. albicans* biofilms in alternative models. In mammals, DYRK inhibitors, including β-carbolines, show promise in treating Alzheimer’s Disease^55^, Down Syndrome^56^, cancer^57^, and diabetes^58^. Through these investigations, a wealth of chemical matter has been generated which remains to be evaluated for activity against fungi. In support of this notion, we showed 1-ECBC a molecule structurally similar to 1-ABC, displays improved stability upon incubation with mammalian cells while also abrogating *C. albicans* biofilm formation in vitro and in vivo. Its activity is similar to that of the small molecule filastatin, which blocks *C. albicans* adhesion, impairs hyphal morphogenesis, and impedes biofilm formation in an ex vivo murine vaginal model, though its target is unknown^34^. While anti-virulence strategies are an emerging area of antifungal drug development that remain largely unexplored^25^, in the realm of bacterial infections, anti-virulence strategies are thought to be effective for both treatment and vaccination efforts^59^. Continued exploration of inter-kingdom microbial interactions provides an exciting approach for leveraging ancient biology to unveil additional treatments to combat the fungal infections that afflict millions of people worldwide.

## Methods

### Growth conditions

All strains used in this study are listed in Supplementary Table 1, all plasmids used for strain construction are listed in Supplementary Table 2, and all oligonucleotide sequences used in this study are listed in Supplementary Table 3. *C. albicans* strains were maintained at −80 °C in rich medium (YPD) with 25% glycerol and *C. albicans* strains were grown in standard conditions in YPD at 30 °C, unless otherwise indicated. YPD was prepared with 1% yeast extract, 2% glucose, 2% bactopeptone and 2% agar for solid medium. Where indicated, strains were grown in the presence of doxycycline (DOX) dissolved in filter-sterilized water (Doxycycline Hydrochloride, BioBasic, DB0889). *Lactobacillus* strains obtained from Lallemand Health Solutions Incorporated (Montreal, QC) were grown under standard anaerobic conditions at 37 °C in MRS broth (Sigma-Aldrich). Bacterial strains from the Navarre murine gut collection were grown under standard anaerobic conditions at 37 °C in MRS broth (Sigma-Aldrich) or LB Broth (BioShop). *Streptococcus danieliae*, *Ligilactobacillus animalis, Limosilactobacillus reuteri, Lactobacillus intestinalis, Lactobacillus gasseri, Lactobacillus johnsonii, Enterococcus* spp. and *Staphylococcus* spp. were grown in MRS. All other bacterial strains were grown in LB broth. The β-carbolines used in this study were 1-ABC (MolPort), 1-ECBC (TCG Lifesciences), harmine (Sigma-Aldrich), harmane (MolPort), norharmane (MolPort), and beta-carboline-3-carboxylic acid methylamide (MolPort) prepared as 20 mM DMSO solutions and stored at −20 °C.

### *Lactobacillus* cell-free conditioned medium

*Lactobacillus-*conditioned media was prepared as described previously^60^. Briefly, *Lactobacillus* strains were grown for 18 h in MRS broth at 37 °C in an anerobic chamber. Cells were harvested by centrifugation at 16,000 × *g* for 5 min. The supernatant was collected and centrifuged again. The final supernatant was passed through a 0.2 µm filter to remove any remaining cells. This solution was stored at 4 °C for use throughout all experiments for a maximum of 4 weeks.

For characterization of the active compound present in the conditioned medium, cell-free conditioned medium was incubated for 1 h at 37 °C with 100 mg/L DNase I, RNase A, or Proteinase K (Sigma-Aldrich) as previously described^60^. Following all treatments, the cell-free extracts and controls were incubated at 100 °C for 10 min to inactivate DNase I, RNase A, or Proteinase K prior to being used in the *C. albicans* filamentation assay. To assess the impact of temperature on the cell-free extract, the *Lactobacillus-*conditioned medium was heated for 10, 30, or 60 min at 100 °C without any additional enzymatic treatment.

### *C. albicans* filamentation assays

Filamentation was induced by sub-culturing an overnight culture of *C. albicans* to an OD_600_ of 0.01 in the relevant inducing cue as described. The inducing cue in all filamentation assays was high temperature (42 °C for 6 h) unless otherwise noted. Media for each cue was prepared as previously described^61^. The cues tested included YPD containing 10% (v/v) heat-inactivated newborn bovine calf serum (NCBS, Gibco # 26010066) for 3 h at 37 °C, 5 mM N-acetylglucosamine (GlcNAc) in YPD at 37 °C for 3 h, Spider medium at 37 °C for 6 h, Lee’s medium at 37 °C for 6 h, and growth in an anerobic chamber at 37 °C for 4 h. High temperature was adopted as the primary inducing cue for most investigations as it avoids any potential for confounding chemical-chemical interactions. For all microscopy, *C. albicans* morphology was assessed using differential interference contrast (DIC) microscopy or using the enhanced green fluorescent protein (EGFP) or dsRed channel on a Zeiss Axio Imager M1 microscope (Carl Zeiss) at the same exposure time. A minimum of two independent fields were captured for each well and images are representative of three technical replicates across two biological replicates.

### Isolation and identification of 1-ABC

Diaion HP-20SS resin (20 g L^−1^) was added to medium collected from cultures of anaerobically grown *L. reuteri* and agitated gently overnight at room temperature. The resin was collected, and metabolites were eluted using a water-methanol gradient on a Flash chromatography system. Bioactive fractions were pooled, dried, and resuspended in acetonitrile and water (1:1) to be further purified on an Alliance (Waters) HPLC using a 4.6x250mm Luna C18 5 µm 100 Å column (Phenomonex). Separation used a mobile phase of (A) water + 0.1% formic acid and (B) acetonitrile + 0.1% formic acid flowing at a rate of 1 mL min^−1^ beginning at a composition of 50% B holding at three minutes and escalating linearly to 85% over 13 min. The structure of 1-ABC was elucidated using NMR spectra (1H, COSY, HMBC, HSQC) acquired on a 700 MHz Agilent DD2 spectrometer and a broadband carbon spectrum acquired on a 500 MHz Agilent DD2 NMR spectrometer equipped with a 13C-sensitive cryogenically cooled probe. MS/MS fragmentation data were collected on a Xevo G2s qToF (Waters) equipped with an electrospray ionization source, isolating the 1-ABC proton adduct by applying 15 eV collision energy.

The production and prevalence of 1-ABC across several *Lactobacillus* isolates was monitored using LC-MS. Strains were grown anaerobically to saturation, bacteria were removed with a 0.2 μm filter and 10 µL of the remaining conditioned media was separated on an Acquity UPLC (Waters) with a 1.7 μm BEH C18 (Waters) 2.1 × 50 mM column using a gradient method (A: Water +0.1% (v/v) formic acid B: Acetonitrile +0.1% (v/v) formic acid | 0-9 min: 20% B − 95% B at 0.125 µl min^−1^). Metabolite masses were detected on an inline Xevo G2s qToF (Waters) mass spectrometer equipped with an electrospray ionization source.

### Whole-genome sequencing

To select for filamentous mutants, 1 × 10^8^ cells from six independent overnights were plated on to YPD plates supplemented with 200 µg/mL NAT and 300 µM 1-ABC. After two days of growth at 42 °C, NAT resistant colonies were selected and re-streaked onto a fresh selection plate. Genomic DNA was isolated from resistant colonies with phenol chloroform using standard protocols. The NexteraXT DNA Sample Preparation Kit was used to prepare the libraries for sequencing following the manufacturer’s instructions (Illumina). AMPure XP beads (Agencourt) were used to purify the libraries and a Bioanalyzer High Sensitivity DNA Chip (Agilent Technologies), and a Qubit High Sensitivity dsDNA fluorometric quantification kit (Life Technologies) were used to quantify the concentration of the library. An Illumina MiSeq sequencing platform was used to sequence the DNA libraries using paired end 2 × 250 flow cells. MuTect (version 1.1.4.)^62^ was used to identify unique mutations in lineages resistant to the filament-repressive effects of 1-ABC. Copy number variation of each sequenced strain was visualized using Y_MAP_ (version 1.0)^63^.

### Quantitative reverse transcription-PCR (qRT-PCR)

Cells were grown overnight in YPD at 30 °C, diluted to an OD_600_ of 0.1, and grown at 30 °C to mid-log phase in 0, 1, or 5 µg/mL DOX. Cultures were pelleted and frozen at −80 °C. RNA extraction, complementary DNA synthesis and PCR were performed as previously described^64^. In brief, PCR was performed using Fast SYBR green master mix (Applied Biosystems) and a Bio-Rad CFX384 real-time system, under the following cycling conditions: 95 °C for 3 min, followed by 95 °C for 10 s and 60 °C for 30 s, for 40 cycles. Reactions were performed in technical quadruplicate for two biological replicates. Data were analyzed using Bio-Rad CFX Manager 3.1. All data were normalized to the GPD1 reference gene for *C. albicans*. Error bars represent standard error of the mean.

### *C. albicans* in vitro biofilm assay

Biofilm assays were performed as previously described^33^. A wild-type strain of *C. albicans* was grown overnight in YPD at 30 °C for 18 h. Cells were then counted using a hemacytometer and re-suspended in RPMI medium buffered with MOPS at a concentration of 1 × 10^6^ cells/mL. In total, 100 µL of the cell suspension was added to each well of a 96-well microtiter plate (Sarstedt) primed with newborn calf bovine serum (Gibco). Plates were incubated at 37 °C for 120 min. Wells were then washed twice with 100 µL phosphate-buffered saline (PBS) to remove any non-adherent cells. Fresh RPMI was then added containing either 1-ECBC (TCG Lifesciences), or solvent only control. Plates were incubated at 37 °C for 24 h, non-adherent cells were washed away with PBS and biofilm metabolic activity was measured via reduction of XTT. Following addition of 90 μL of XTT (X4251, Sigma) at 0.5 mg/mL and 10 μL phenazine methosulfate (P9625, Sigma) at 320 μg/mL, plates were incubated at 37 °C for 10 min. The supernatant was transferred to a fresh plate and its absorbance was measured at 490 nm using an automated plate reader (Sarstedt). Experiments conducted with five technical replicates per condition in biological duplicate.

### *C. albicans* viability assay

To visualize cell death, propidium iodide staining was visualized using fluorescence microscopy. Briefly, cells were sub-cultured from a saturated overnight culture to an OD_600_ of 0.1 in YPD medium and grown for 18 h at 42 °C in the absence or presence of 30% v/v *L. reuteri-*conditioned medium or lactic acid. A yeast control was grown for 18 h at 30 °C and ethanol controls of either yeast or filamentous cultures were prepared by incubating *C. albicans* with 70% ethanol for 30 min prior to microscopy. For imaging, cells were pelleted and resuspended in PBS with 1 µg/mL propidium iodide. Cells were imaged using differential interference contrast (DIC) microscopy and using the dsRed channel on a Zeiss Axio Imager.MI microscope (Carl Zeiss) at the same exposure time.

For *C. albicans* growth curves, strains were grown in YPD medium overnight and diluted to an OD_600_ of 0.0001 in YPD medium in 96-well plates to a final volume of 100 μL. Plates were grown at 30 °C in shaking conditions and OD_600_ was measured every 15 min for 24 h using a TECAN GENios instrument.

### Flow cytometric analysis of *C. albicans* filamentation

A CytoFlex flow cytometer (Beckman Coulter) was used to quantify fluorescence of an *pHWP1-*GFP strain of *C. albicans*. Briefly, *C. albicans* cells were sub-cultured from a saturated overnight culture to an OD_600_ of 0.1 in YPD medium and grown for 6 h at 42 °C in the absence or presence of 1-ABC or *Lactobacillus*-conditioned medium. Cells were then pelleted, washed once with PBS, resuspended in PBS, and diluted 1:10 in 1 mL PBS. 250 µL of each sample were added to a flat-bottom transparent 96-well plate (Beckman Coulter). Samples were run using the CytExpert software (version 2.4) until 10,000 events were recorded. Populations were gated to exclude debris and the median  Channel 1 (FL-1) fluorescence value was taken for each sample to determine the median brightness of each cell in each sample.

### Compound stability in human cell culture

Commercially available β-carbolines were incubated overnight with a monolayer of human HepG2 cells in DMEM + 10% FBS. Following overnight incubation, the plate was spun down, and supernatant was gently removed. Supernatant was combined in a 50% (v/v) combination with YPD containing OD_600_ = 0.1 wild type *C. albicans* (SN95). Light microscopy was used to assess the *C. albicans* filamentation phenotype after growth at 37 °C with 5% FBS for 6 h. Compounds that were inactivated by the HepG2 monolayer did not block filamentation, whereas compounds that were not inactivated were able to block the filamentation phenotype.

### Murine vaginal explant biofilm model

Mouse tissues were acquired and analyzed using protocols approved by the Institutional Animal Care and Use Committee (IACUC) of Louisiana State University Health Sciences Center (Protocol 3663). All mice were maintained at 22 °C, 45% humidity, and with light/dark alternating every 12 h. The protocol for the vaginal explant model was performed as previously described^65^. In brief, female mice were administered estrogen (1 μg/mL; 100 μL subQ) for 72 h prior to harvesting of vaginas. Tissues were inoculated with 100 μL *C. albicans* 96113 at 2.5 × 10^4^ cells/mL in acetate buffer containing 0 mM, 1 mM, or 5 mM 1-ECBC. After 24 h of growth at 37 °C, tissues were bisected, and one half was used for CFU analysis and the other half was processed and stained with Calcofluor white (2.5 μg/mL) or Concavalin A-Texas Red conjugate (50 μg/mL) for confocal microscopy. Confocal microscopy images were taken at ×600 magnification. LDH release was detected in the tissue hydration solution (water) at 24-h post-inoculation using an LDH assay kit (Abcam, Inc.). Two independent experiments were performed with 3 vaginal explants per experiment.

### Rat indwelling catheter biofilm model

All animal procedures were approved by the Institutional Animal Care and Use Committee at the University of Wisconsin-Madison according to the guidelines of the Animal Welfare Act, The Institute of Laboratory Animals Resources Guide for the Care and Use of Laboratory Animals, and Public Health Service Policy. The approved animal protocol number is DA0031. All rats were maintained at 22.2 °C, 45% humidity, and with light/dark alternating every 12 h. The protocol for the rat catheter biofilm was conducted as previously described^35^. In brief, indwelling central venous catheters in rats were inoculated with 1 × 10^6^ cells/mL *C. albicans* with or without 1-ECBC (400 μM). To quantitate fungal burden after 24 h, the catheter was removed, and the tip placed in a one milliliter of sterile 0.85% NaCl. A 1:10 serial dilution of this wash fluid was plated on SDA and macroscopic fungal colonies counted after 24 h of growth at 35 °C^66^. For microscopy, catheters were fixed overnight in 4% formaldehyde and 1% glutaraldehyde in PBS. The catheters were then washed with PBS and treated with 1% osmium tetroxide in PBS for 30 min. Alcohol washes were used to dry the segments before they were mounted, and gold coated. All images were taken using a Zeiss GeminiSEM 450 at 3 kV.

### Immunoprecipitation of 6XHis-3XFlag-tagged Yak1 and kinase assay

Approximately 40 grams of cells (CaLC7453; wet weight) were collected from 4 L of a mid-late log phase culture grown in YPD. The cell pellet was lysed by grinding in liquid nitrogen for 30 min using a mortar and pestle. The resulting cell powder was dissolved in 80 mL of 1.5X lysis buffer (50 mM HEPES-NaOH pH 7.2, 300 mM NaCl, 1.5 mM EGTA, 15 mM NaF, 15% (v/v) glycerol). The lysis buffer was supplemented with protease (1 mM PMSF, 2 mM benzamidine HCl, 0.6 μM leupeptin, 2 μM pepstatin A) and phosphatase inhibitors (2 mM sodium vanadate, 2 mM sodium pyrophosphate,5 mM β-glycerophosphate). Next the sample was sonicated at 30% amplitude for 10 s on and 10 s off for a total of 5 min (Misonix S-4000 dual horn with 3/4 -inch probes). Magnesium (II) chloride and IGEPAL-CA630 were then added to a final concentration of 2 mM and 0.02% respectively. The suspension was treated with 300 U benzonase (Millipore) for 15 min at 4 °C with gentle stirring. Insoluble materials were removed by ultracentrifugation at 125,000 × *g* in a Type 45 Ti rotor (Beckman) for 90 min at 4 °C. The clarified cell lysate was incubated with 1.6 mL of anti-Flag affinity gel (Sigma) in a batch format for 90 min at 4 °C. Gel beads were washed three times by 8 bead volumes of wash buffer (25 mM HEPES-NaOH pH 7.2, 200 mM NaCl, 0.02 % IGEPAL-CA630, 10% glycerol) supplemented with 2 mM NaF and the other inhibitors used in the lysis buffer, and then by the wash buffer without inhibitors. Immobilized Yak1 was eluted by incubating the gel beads with wash buffer containing 100 µg/mL 3xFlag peptide. Multiple fractions were collected with a 15 min incubation between fractions. The kinase activity of the peak fraction was tested using the ADP-Glo assay kit (Promega) in 10 µL reactions which contained 12.5 mM HEPES-NaOH pH 7.2, 100 mM NaCl, 0.01% (v/v) IGEPAL-CA630, 5% (v/v) glycerol, and 2.5 mM MgCl_2_ with or without 0.2 mg/mL DYRKtide (SignalChem). β-carbolines were added to achieve each indicated concentration using a Tecan D300e compound dispenser, and then ATP was added to a final concentration of 10 µM to initiate the reaction. After 40 min, the reaction was stopped by incubation at 30 °C. Luminescence was measured using a TECAN SPARK plate reader in a 384-well plate.

### Computational model of 1-ECBC binding Yak1

Mammalian DYRK2 (PDB 3KVW) was used as a template to homology model the structure of the *C. albicans* Yak1 kinase domain using Phyre2 (version 2.0)^67^, which was energy minimized using YASARA (version 20,12,24)^68^. This model was then used for docking of 1-ECBC into the ATP-binding site of the kinase domain using Autodock Vina (version 1.1.2.)^69^. The model was visualized using PyMOL (version 2.5.0.).

### Strain construction

CaLC7195: CaLC3900 (*pHWP1*-NAT) was grown at 42 °C for 72 h in the presence of 200 µg/mL NAT and 300 µM 1-ABC. Whole-genome sequencing identified a loss of heterozygosity (LOH) substitution (Oca6^I43^/Oca6^I43^). The LOH event was confirmed via Sanger sequencing by amplifying the ORF using oLC8740 and oLC8741 and using oLC8730 as the sequencing primer.

CaLC7196: CaLC3900 (*pHWP1*-NAT) was grown as described for CaLC7195. Whole-genome sequencing identified a single nucleotide polymorphism substitution (Rob1^L672V^/Rob1). The SNP was confirmed via Sanger sequencing by amplifying the ORF using oLC2637 and oLC8759 and using oLC8303 as the sequencing primer.

CaLC7197: CaLC3900 (*pHWP1*-NAT) was grown as described for CaLC7195. Whole-genome sequencing identified a loss of heterozygosity (LOH) substitution (Acp1^I19^/ Acp1^I19^). The LOH event was confirmed via Sanger sequencing by amplifying the ORF using oLC8746 and oLC8747 and using oLC8724 as the sequencing primer.

CaLC7198: CaLC3900 (*pHWP1*-NAT) was grown as described for CaLC7195. Whole-genome sequencing identified a loss of heterozygosity substitution (orf19.1831^A124^/orf19.1831^A124^). The LOH event was confirmed via Sanger sequencing by amplifying the ORF using oLC8896 and oLC8701 and using oLC8931 as the sequencing primer.

CaLC7199: CaLC3900 (*pHWP1*-NAT) was as described for CaLC7195. Whole-genome sequencing identified a loss of heterozygosity substitution (orf19.3728^T222^/orf19.3728^T222^). The LOH event was confirmed via Sanger sequencing by amplifying the ORF using oLC8750 and oLC8751 and using oLC8708 as the sequencing primer.

CaLC7200: CaLC3900 (*pHWP1*-NAT) was grown as described for CaLC7195. Whole-genome sequencing identified a loss of heterozygosity substitution (Yku80^I152^/ Yku80^I152^). The LOH event was confirmed via Sanger sequencing by amplifying the ORF using oLC8756 and oLC8757 and using oLC8736 as the sequencing primer.

CaLC7201: CaLC7195 was transformed with a PCR fusion product of wild type *OCA6* fused to *ARG4*. The wild type *OCA6* allele was amplified with primers oLC8740 and oLC8916. The reverse primer (oLC8916) contained 20 bp of homology to the 5ʹ-end of the *ARG4* gene. *ARG4* was then amplified with oLC8917 and oLC8918. The forward primer (oLC8917) contained 20-bp of homology to the 3ʹ-end of *OCA6* and the reverse primer (oLC8918) contained 70-bp of homology of the 3ʹ-UTR of *OCA6* to facilitate homologous recombination. Transformants were selected by plating cells onto medium lacking arginine. To confirm integration of the *ARG4* cassette, primers oLC8730 and oLC8916 were used to amplify the *OCA6* cassette. Correct integration of wild type *OCA6* was confirmed via Sanger sequencing by amplifying the ORF using oLC8740 and oLC8741 and using oLC8730 as the sequencing primer.

CaLC7202: CaLC3900 was transformed with a PCR fusion product of mutant *OCA6* from lineage 1 fused to *ARG4*. The mutant *OCA6* allele was amplified from CaLC7195 with primers oLC8740 and oLC8916. The reverse primer (oLC8916) contained 20 bp of homology to the 5ʹ-end of *ARG4*. *ARG4* was then amplified with oLC8917 and oLC8918. The forward primer (oLC8917) contained 20-bp of homology to the 3ʹ-end of *OCA6* and the reverse primer (oLC8918) contained 70-bp of homology of the 3ʹ-UTR of *OCA6* to facilitate homologous recombination. Transformants were selected by plating cells onto medium lacking arginine. To confirm integration of the *ARG4* cassette, primers oLC8730 and oLC8916 were used to amplify the *OCA6* cassette. Correct integration of mutant *OCA6* was confirmed via Sanger sequencing by amplifying the ORF using oLC8740 and oLC8741 and using oLC8730 as the sequencing primer.

CaLC7203: CaLC7196 was transformed with a PCR fusion product of wild type *ROB1* fused to *ARG4*. The wild-type *ROB1* allele was amplified from CaLC3900 with primers oLC2888 and oLC8906. The reverse primer (oLC8906) contained 20-bp of homology to the 5ʹ-end of *ARG4*. *ARG4* was amplified with oLC8907 and oLC8908. The forward primer (oLC8907) contained 20- bp of homology to the 3ʹ-end of *OCA6* and the reverse primer (oLC8908) contained 70-bp of homology of the 3 -UTR of *OCA6* to facilitate homologous recombination. Transformants were selected by plating cells onto medium lacking arginine. To confirm integration of the *ARG4* cassette, primers oLC5570 and oLC8759 were used to amplify the *ROB1* cassette. Correct integration of wild type *ROB1* was confirmed via Sanger sequencing by amplifying the ORF using oLC2637 and oLC8759 and using oLC8303 as the sequencing primer.

CaLC7285: CaLC3900 was transformed with a PCR fusion product of mutant *ROB1* from CaLC7196 fused to *ARG4*. *ROB1* was amplified from CaLC7196 with primers oLC2888 and oLC8906. The reverse primer (oLC8906) contained 20-bp of homology to the 5ʹend of *ARG4*. *ARG4* was amplified with oLC8907 and oLC8908. The forward primer (oLC8907) contained 20- bp of homology to the 3ʹ-end of *ROB1* and the reverse primer (oLC8908) contained 70-bp of homology of the 3ʹ-UTR of *ROB1* to facilitate homologous recombination. Transformants were selected by plating cells onto medium lacking arginine. To confirm integration of the *ARG4* cassette, primers oLC5570 and oLC8759 were used to amplify the *ROB1* cassette. Correct integration of mutant *ROB1* was confirmed via Sanger sequencing by amplifying the ORF using oLC2637 and oLC8759 and using oLC8303 as the sequencing primer.

CaLC7286: *YAK1* in evolved lineage 1 (CaLC7195) was deleted by a transient CRISPR method (Min, et al., 2016). The guide RNA was generated using a fusion PCR strategy. The first component for the fusion PCR product was generated from pLC1081 using oLC9199 and oLC6926. The second component for the fusion PCR product was generated from pLC1081 using oLC9200 and oLC6927. The fusion PCR product was then generated using nesting primers oLC6928 and oLC6929. The repairing template was amplified from pLC1100 using oLC9201 and oLC9203. oLC9204 and oLC9205 and oLC9206 and oLC9207 were used to verify the absence of *YAK1*.

CaLC7287: *YAK1* in evolved lineage 2 (CaLC7196) was deleted as described for CaLC7286.

CaLC7453: Both alleles of *YAK1* were C-terminally tagged with 6X His-3X. Primers oLC9512 and oLC9513 were used to amplify the tag from pLC1031. The amplicon was introduced into YAK1 loci in CaLC7426 by a transient CRISPR method. sgRNA was generated by YAK1 specific primers oLC9452 and oLC9453 as well as universal primers oLC6926, oLC6927, oLC6928, and oLC6929 from pLC1081. Correct integration was checked using oLC4714 and oLC9205 and the absence of the native YAK1 promoter-ORF junction was confirmed using oLC6393 and oLC9205. Overexpression of Yak1-HF was confirmed by immunoblotting.

### Reporting summary

Further information on research design is available in the Nature Research Reporting Summary linked to this article.

### Supplementary information

Supplementary Information

Reporting Summary

### Source data

Source Data

## Data Availability

Source data are provided with this paper. All additional data, including raw data and images associated with all figures, is available from the corresponding author, L.E.C, upon reasonable request. Source data are provided with this paper.
