# Addendum: A small molecule produced by *Lactobacillus* species blocks *Candida albicans* filamentation by inhibiting a DYRK1-family kinase

**DOI:** 10.1038/s41467-024-50740-z

**Published:** 2024-08-01

**Authors:** Jessie MacAlpine, Martin Daniel-Ivad, Zhongle Liu, Junko Yano, Nicole M. Revie, Robert T. Todd, Peter J. Stogios, Hiram Sanchez, Teresa R. O’Meara, Thomas A. Tompkins, Alexei Savchenko, Anna Selmecki, Amanda O. Veri, David R. Andes, Paul L. Fidel, Nicole Robbins, Justin Nodwell, Luke Whitesell, Leah E. Cowen

**Affiliations:** 1 Department of Molecular Genetics, University of Toronto, Toronto, ON Canada; 2 Department of Biochemistry, University of Toronto, Toronto, ON Canada; 3 Center of Excellence in Oral and Craniofacial Biology, Louisiana State University Health Sciences Center School of Dentistry, New Orleans, LA USA; 4 Department of Microbiology and Immunology, University of Minnesota Medical School, Minneapolis, MN USA; 5 BioZone, Department of Chemical Engineering and Applied Chemistry, University of Toronto, Toronto, ON Canada; 6 Center for Structural Genomics of Infectious Diseases (CSGID), Chicago, IL USA; 7 Department of Medicine, University of Wisconsin School of Medicine and Public Health, Madison, WI USA; 8 Department of Medical Microbiology and Immunology, University of Wisconsin, Madison, WI USA; 9 Department of Microbiology and Immunology, University of Michigan Medical School, Ann Arbor, MI 48109 USA; 10 Rosell Institute for Microbiome and Probiotics, 6100 Avenue Royalmount, Montreal, QC Canada; 11 Department of Microbiology, Immunology and Infectious Diseases, University of Calgary, Calgary, AB Canada

Addendum to: *Nature Communications* 10.1038/s41467-021-26390-w, published online 22 October 2021

In the original version of the article^1^, we reported that *Lactobacillus*-conditioned MRS medium and laboratory-synthesized 1-acetyl-beta-carboline (1-ABC) inhibit a key virulence trait in the human fungal pathogen *Candida albicans*, namely its ability to undergo yeast-to-hyphal morphogenetic transition. Herraiz et al.^2^ subsequently discovered that the presence of 1-ABC in *Lactobacillus*-conditioned medium is not due to bacterial biosynthesis of the compound, but is instead the result of spontaneous chemical reactions that occur in MRS medium under the high pressure and heat that accompany the autoclave sterilization process.

In light of these findings, we conducted follow-up experiments—summarized in our Reply^3^—that confirmed that 1-ABC is spontaneously generated during the autoclaving of glucose-containing MRS. However, the concentration of 1-ABC in the medium is not sufficient to block morphogenesis of *C. albicans* without subsequent acidification of the MRS as occurs during the growth of lactobacilli in it. Apparently, our bioassay-guided small-molecule enrichment and isolation process yielded fractions with 1-ABC at concentrations sufficient to block *C. albicans* morphogenesis, a result that was confirmed with pure, synthetic 1-ABC.

In summary, the generation of 1-ABC during autoclaving in combination with acidification of the culture medium during growth of lactobacilli led us to misinterpret results and erroneously conclude that lactobacilli synthesize 1-ABC. However, all other findings in the original version of the article^1^, related to the mode of action and therapeutic potential of 1-ABC, remain valid as the experiments were conducted with pure compound.
