# Supplementary material for: A small molecule produced by Lactobacillus species blocks Candida albicans filamentation by inhibiting a DYRK1-family kinase
Source: Nat Commun. 2021 Oct 22;12:6151. doi: 10.1038/s41467-021-26390-w (PMC8536679; doi:10.1038/s41467-021-26390-w)
Supplement: Supplementary file 1 — Supplementary Information [file 41467_2021_26390_MOESM1_ESM.pdf]

A Small Molecule Produced by *Lactobacillus* Species Blocks *Candida albicans* Filamentation by Inhibiting a DYRK1-Family Kinase

SUPPLEMENTARY FIGURES

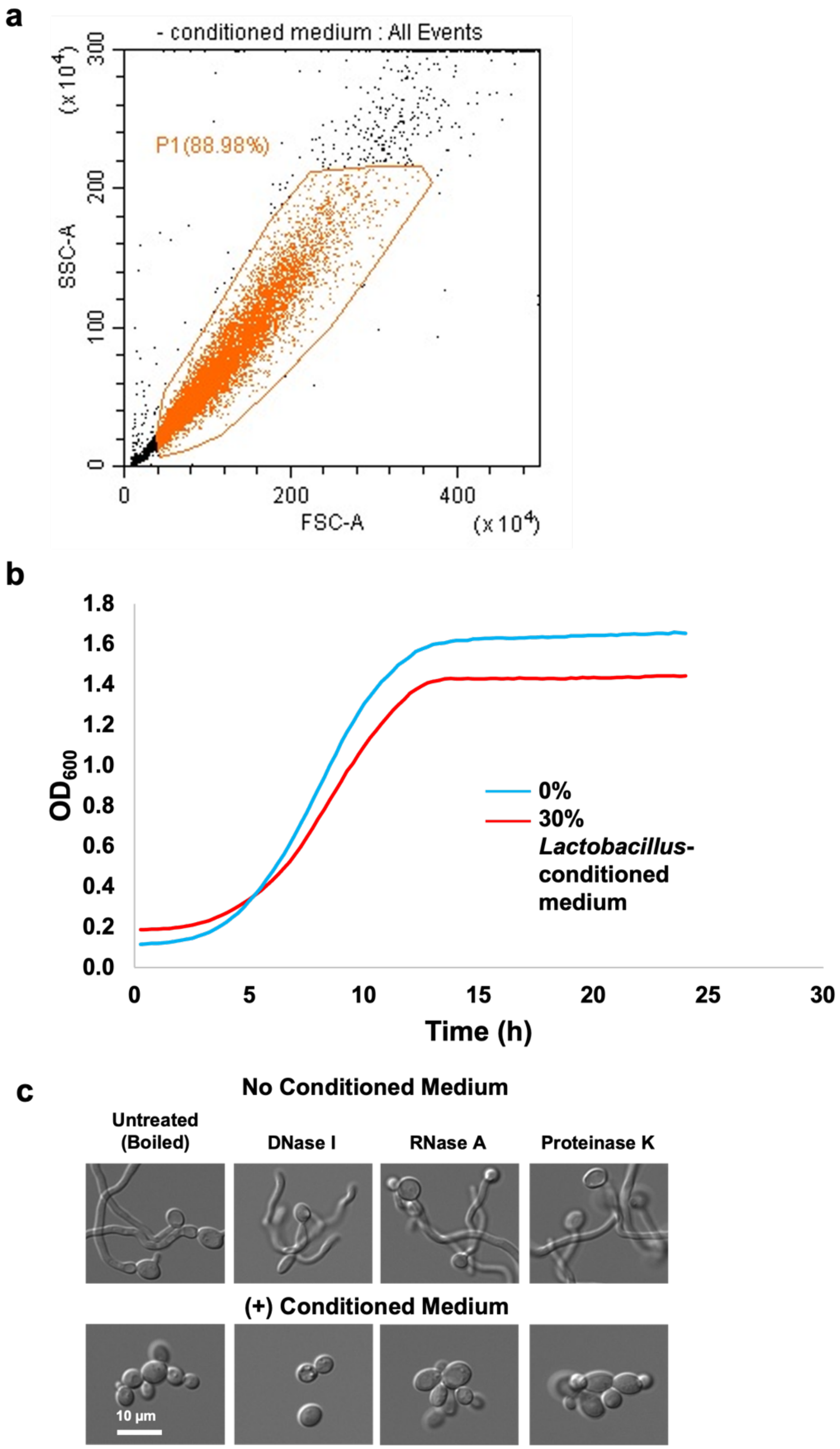

Supplementary Fig. 1: Flow cytometry used to quantify *C. albicans* filamentation. a) Gating strategy used for flow cytometry experiments involving *pHWP1-GFP C. albicans.* **b)** Growth

curve of wild-type *C. albicans* cultured in the presence of 0% or 30% v/v *Lactobacillus*-conditioned medium for 24 hours at 30°C under shaking conditions. Growth was assessed by measuring OD_{600} every 15 minutes for 24 hours. Source data are provided as a Source Data file. **c)** DNase I, RNase A, or Proteinase K treatment (100 mg/L, one hour at 37℃) did not impair the filamentation-repressing activity of *L. rhamnosus*-conditioned medium (30% v/v). After treatment, media were added to cultures of *C. albicans* and cells were imaged after incubation at 42℃ for 6 hours. Data represents two biological replicates.

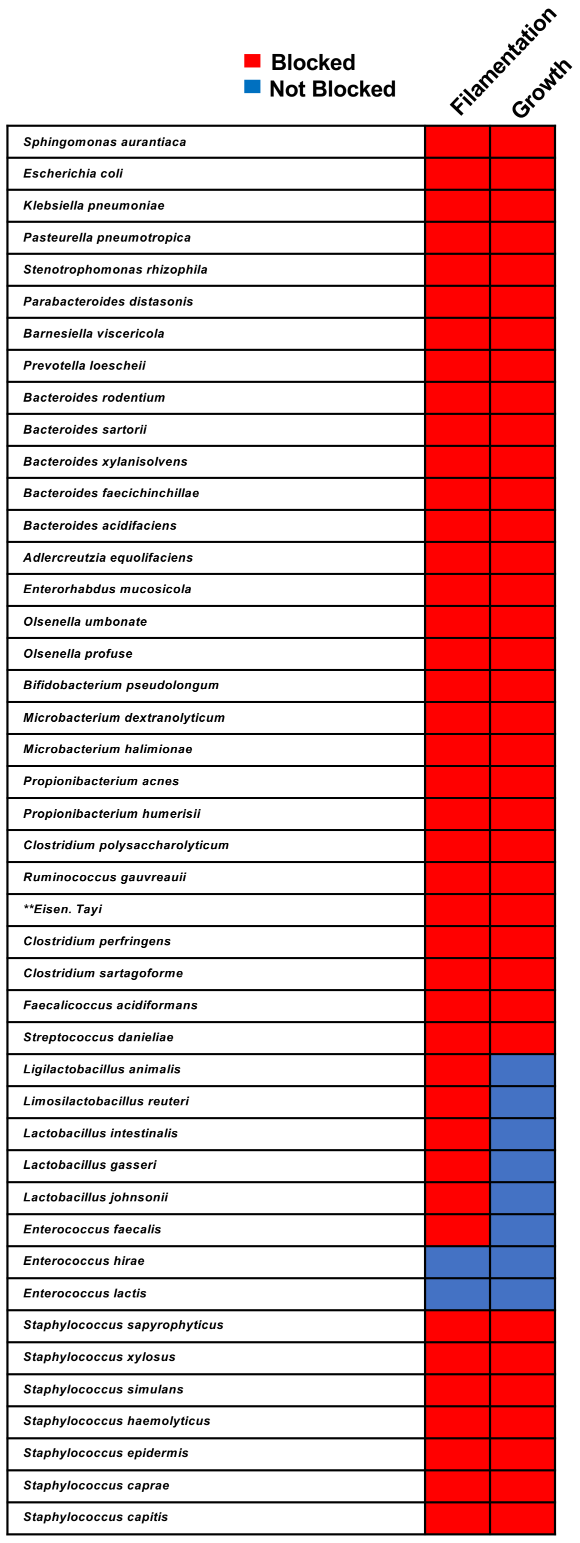

Supplementary Fig. 2: Select *Lactobacillus* species and *Enterococcus faecalis* inhibit *C.*

***albicans* hyphal morphogenesis without affecting growth.** Conditioned-medium (30% v/v) from 44 bacterial isolates from the mouse gut and nine industrial *Lactobacillus* isolates were tested for their ability to block *C. albicans* filamentation in response to high temperature (42℃, 6 hours). Growth was assessed qualitatively by light microscopy. Red indicates a block in filamentation or impaired growth. Blue represents filamentous growth and robust growth.

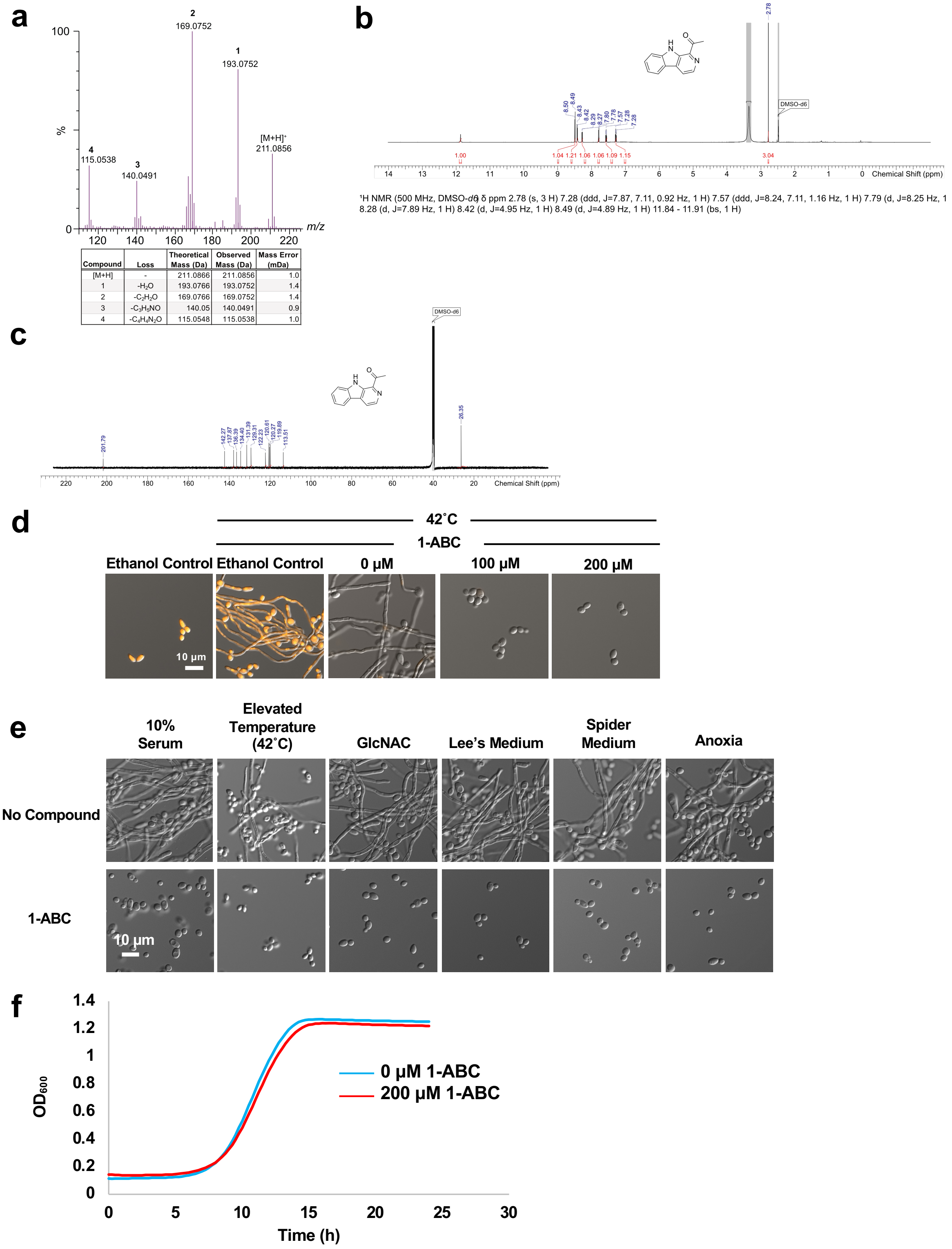

Compound	Loss	Theoretical Mass (Da)	Observed Mass (Da)	Mass Error (mDa)
[M+H]	-	211.0866	211.0856	1.0
1	-H_2O	193.0766	193.0752	1.4
2	-C_2H_2O	169.0766	169.0752	1.4
3	-C_3H_5NO	140.05	140.0491	0.9
4	-$C_4H_4N_2O$	115.0548	115.0538	1.0

^{1}H NMR (500 MHz, DMSO-*d6*) δ ppm 2.78 (s, 3 H) 7.28 (ddd, J=7.87, 7.11, 0.92 Hz, 1 H) 7.57 (ddd, J=8.24, 7.11, 1.16 Hz, 1 H) 7.79 (d, J=8.25 Hz, 1 8.28 (d, J=7.89 Hz, 1 H) 8.42 (d, J=4.95 Hz, 1 H) 8.49 (d, J=4.89 Hz, 1 H) 11.84 - 11.91 (bs, 1 H)

Supplementary Fig. 3: Identification of *Lactobacillus*-secreted 1-ABC. a) Tandem mass

spectrometry fingerprint of 1-ABC conforms to the elucidated structure of 1-ABC. b) 1H NMR Spectrum for 1-ABC in d6-DMSO. c) 13C NMR Spectrum for 1-ABC in d6-DMSO. d) 1-ABC treatment did not affect *C. albicans* viability, as indicated by propidium iodide staining. Aliquots of cells were treated with 70% ethanol to serve as a positive control for cell death and fluorescence was visualized via microscopy. Data represent two biological replicates. e) 1-ABC blocks *C. albicans* filamentation in response to diverse inducing cues including elevated temperature (42℃, 6 hours, YPD), 10% serum (37℃, 3 hours, YPD), 5 mM N-acetylglucosamine (GlcNAc; 37℃, 3 hours, YPD), Lee's Medium (37°C, 6 hours), Spider Medium (37℃, 6 hours), and growth under anerobic conditions (37℃, 4 hours, YPD). Data represent two biological replicates. f) Growth curve of wild-type *C. albicans* cultured in the presence of 0 μM or 200 μM 1-ABC for 24 hours at 30°C under shaking conditions. Growth was assessed by measuring OD_{600} every 15 minutes for 24 hours. Source data are provided as a Source Data file.

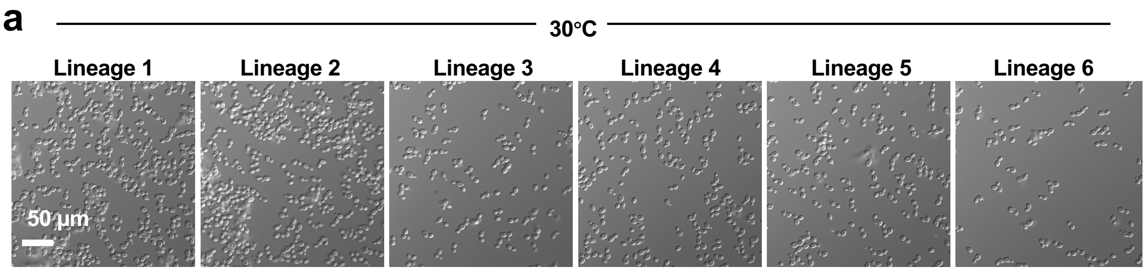

b

Lineage	Gene	Description	Substitution	Type of Mutation
3	*ACP1*	Mitochondrial acyl carrier protein involved in fatty acid biosynthesis	M19I	LOH
4	*orf19.1831*	Unknown function	T124A	LOH
5	*orf19.3728*	Predicted phosphatase binding and regulation	N222T	LOH
6	*YKU80*	NHEJ double strand break repair	L152I	LOH

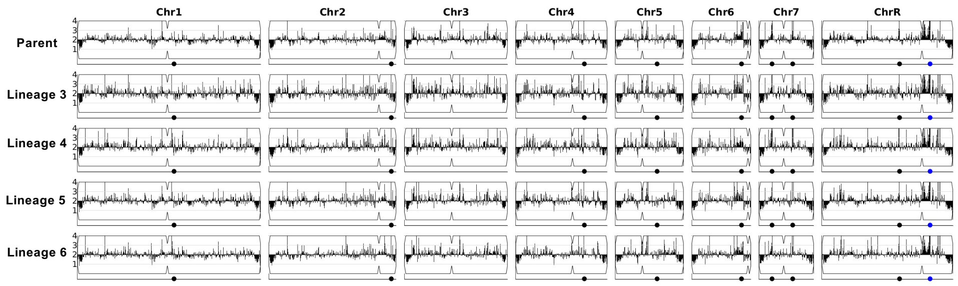

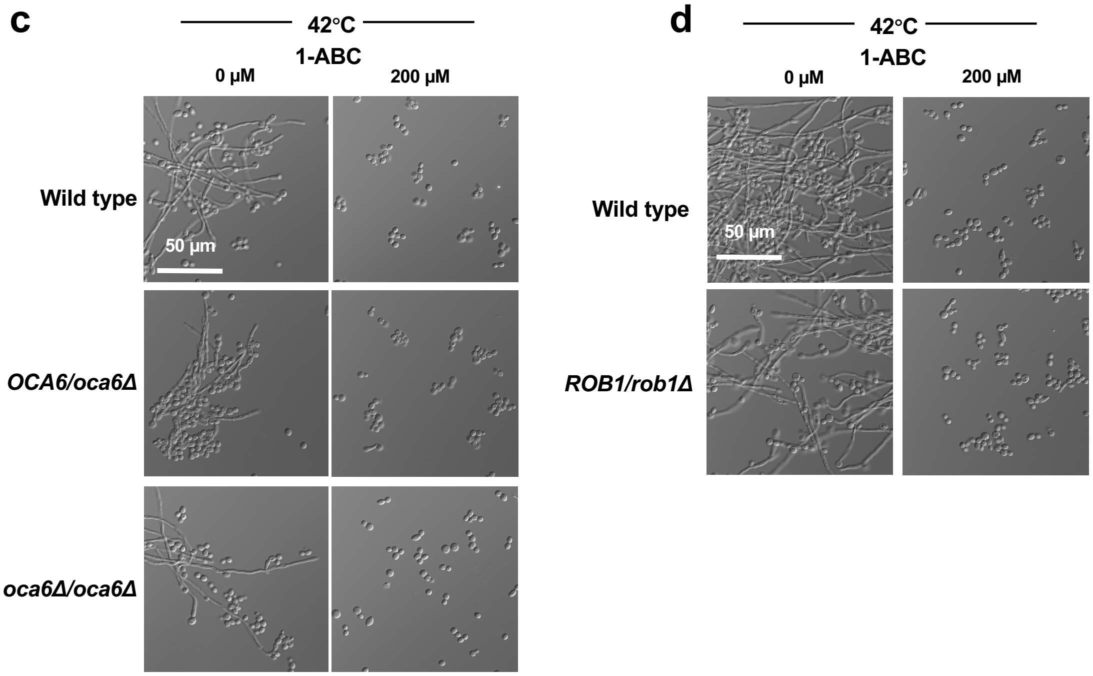

Supplementary Fig. 4: Selection-based strategy identifies six independent mutants with

restored filamentation in the presence of 1-ABC. a) The six lineages capable of filamenting in response to 1-ABC do not exhibit constitutive filamentation. Cultures were grown at 30°C for 18 hours. Data represents three biological replicates. b) Whole-genome sequencing analysis of all six mutant lineages did not detect any copy number variants in the mutants relative to the parental strain. Copy number variant analysis was conducted and visualized using the Y-MAP software[1]. Summary of whole-genome sequencing results for Lineages 3-6. All identified loss-of-heterozygosity events or single nucleotide polymorphisms that occurred within open reading frames are shown. Results were independently validated using Sanger sequencing. c) Deletion of *OCA6* does not affect the *C. albicans* response to 1-ABC. Filamentation was induced by growth at elevated temperature (42°C, 6 hours). Data represent three biological replicates. d) Heterozygous deletion of *ROB1* does not affect the *C. albicans* response to 1-ABC. Filamentation was induced by growth at elevated temperature (42°C, 6 hours). Data represent three biological replicates.

a

Compound	Structure	Minimum Effective Concentration	Inactivation by HepG2
1-ABC	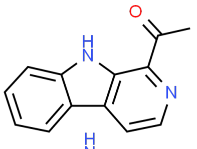	25 µM	YES
Beta-carboline-3-carboxylic acid methylamide	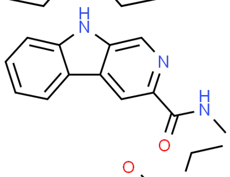	100 µM	YES
1-ECBC	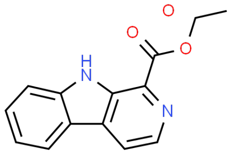	50 µM	NO
Harmane	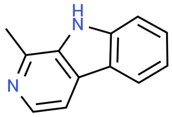	200 µM	YES
Norharmane	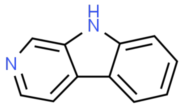	>200 µM	YES
Harmine	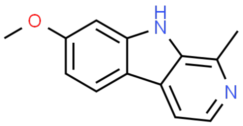	200 µM	N/A

b

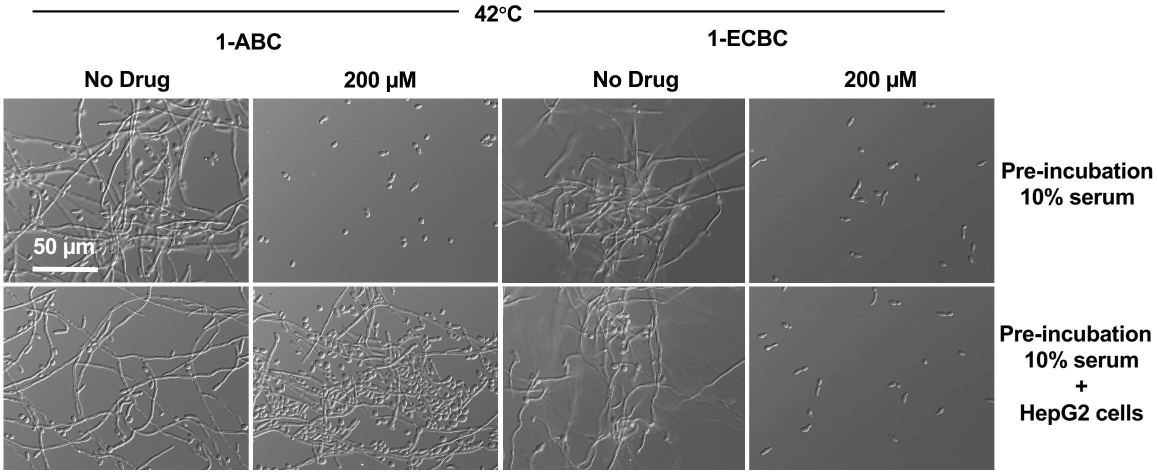

Supplementary Fig. 5: 1-ECBC inhibits *C. albicans* filamentation and is not inactivated by

human cells. a) Bioactivity and stability data for β -carbolines as monitored by ability to block *C. albicans* filamentation induced by 5% serum at 37℃ for six hours. b) 1-ABC was inactivated by human HepG2 cells following incubation with a monolayer of cells overnight, while the alternative β-carboline, 1-ECBC, retained activity. *C. albicans* filamentation was induced with 5% serum at 37℃ for six hours. Light microscopy images were taken at 10X magnification. Data represent two biological replicates.

SUPPLEMENTARY TABLES

Supplementary Table 1: Strains used in this study.

**Accession Number**	**Strain Name**	**Genotype**	**Source**
CaLC239	SN95	*arg4Δ/arg4Δ his1Δ/his1Δ URA3/ura3::imm434 IRO1/iro1::imm434*	[2]
CaLC715	*HWP1p-GFP/HWP1*		[3]
CaLC1724	GRACE-1198	*ura3::imm434/ura3::imm434 his3::HISG/his::HISGtetR-GAL4AD::URA3 yak1::HIS3 SAT1-tetp-YAK1*	[4]
CaLC3365	CaSS1	*ura3::imm434/ura3::imm434 his3::HISG/his::HISGtetR-GAL4AD::URA3*	[4]
CaLC3900	*HWP1p-NAT-HIS/HWP1*	*arg4Δ/arg4Δ his1Δ/his1Δ URA3/ura3::imm434 IRO1/iro1::imm434 HWP1p-NAT-HIS/HWP1*	[5]

LrLC4744	*Lacticaseibacillus rhamnosus* R0011	Wild type	Lallemand Health Solutions Inc.
LhLC4745	*Lactobacillus helveticus* R0052	Wild type	Lallemand Health Solutions Inc.
LcLC4746	*Lacticaseibacillus casei* HA108	Wild type	Lallemand Health Solutions Inc.
LrLC4747	*Lacticaseibacillus rhamnosus* HA111	Wild type	Lallemand Health Solutions Inc.
LrLC4748	*Lacticaseibacillus rhamnosus* HA114	Wild type	Lallemand Health Solutions Inc.
LbLC4749	*Levilactobacillus brevis* HA112	Wild type	Lallemand Health Solutions Inc.
LrLC4750	*Limosilactobacillus reuteri* HA118	Wild type	Lallemand Health Solutions Inc.
LpLC4751	*Lactiplantibacillus plantarum* HA119	Wild type	Lallemand Health Solutions Inc.
CaLC7195	Evolved Lineage 1	*arg4Δ/arg4Δ his1Δ/his1Δ URA3/ura3::imm434 IRO1/iro1::imm434 HWP1p-NAT-HIS/HWP1 OCA6I43/OCA6 I43*	This Study

CaLC7196	Evolved Lineage 2	*arg4Δ/arg4Δ his1Δ/his1Δ URA3/ura3::imm434 IRO1/iro1::imm434 HWP1p-NAT-HIS/HWP1 ROB1L672V/ROB1*	This Study
CaLC7197	Evolved Lineage 3	*arg4Δ/arg4Δ his1Δ/his1Δ URA3/ura3::imm434 IRO1/iro1::imm434 HWP1p-NAT-HIS/HWP1 ROB1L672V/ROB1*	This Study
CaLC7198	Evolved Lineage 4	*arg4Δ/arg4Δ his1Δ/his1Δ URA3/ura3::imm434 IRO1/iro1::imm434 HWP1p-NAT-HIS/HWP1 orf19.1831A124/orf19.1831A124*	This Study
CaLC7199	Evolved Lineage 5	*arg4Δ/arg4Δ his1Δ/his1Δ URA3/ura3::imm434 IRO1/iro1::imm434 HWP1p-NAT-HIS/HWP1 orf19.3728T222/orf19.3728T222*	This Study
CaLC7200	Evolved Lineage 6	*arg4Δ/arg4Δ his1Δ/his1Δ URA3/ura3::imm434 IRO1/iro1::imm434 HWP1p-NAT-HIS/HWP1 YKU80I152/YKU80I152*	This Study
CaLC7201	As CaLC7195, Oca6^{N43}/Oca6^{I43}	*his1Δ/his1Δ URA3/ura3::imm434 IRO1/iro1::imm434 HWP1p-NAT-HIS/HWP1 OCA6N43::ARG/OCA6 I43*	This Study
CaLC7202	As CaLC3900, Oca6^{I43}/Oca6^{I43}	*his1Δ/his1Δ URA3/ura3::imm434 IRO1/iro1::imm434 HWP1p-NAT-HIS/HWP1 OCA6I43::ARG/OCA6I43::ARG*	This Study

CaLC7203	As CaLC7196, *ROB1/ROB1*	*his1Δ/his1Δ URA3/ura3::imm434 IRO1/iro1::imm434 HWP1p-NAT-HIS/HWP1 ROB1::ARG/ROB1*	This Study
CaLC7285	As CaLC3900, *ROB1/ROB1^{L672V}*	*his1Δ/his1Δ URA3/ura3 ::imm434 IRO1/iro1 ::imm434 HWP1p-NAT-HIS/HWP1 ROB1/ROB1L672V::ARG*	This Study
CaLC7286	As CaLC7195, *yak1Δ/yak1Δ*	*his1Δ/his1Δ URA3/ura3 ::imm434 IRO1/iro1 ::imm434 HWP1p-NAT-HIS/HWP1 OCA6I43/OCA6I43 yak1::ARG/yak1::ARG*	This Study
CaLC7287	As CaLC7196, *yak1Δ/yak1Δ*	*his1Δ/his1Δ URA3/ura3 ::imm434 IRO1/iro1 ::imm434 HWP1p-NAT-HIS/HWP1 ROB1/ROB1L672V yak1::ARG/yak1::ARG*	This Study
CaLC7453	As CaLC3365, tetO-*YAK1-HIS3*-FLAG/ tetO-*YAK1-HIS3*-FLAG	*ura3::imm434/ura3::imm434 his3::HISG/his::HISGtetR-GAL4AD::URA3 HygB-TAR-tetO-YAK1-6his3Flag-SAT1/HygB-TAR-tetO-YAK1-6his3Flag-SAT1*	This Study

Supplementary Table 2: Plasmids used in this study.

**Plasmid**	**Description**	**Source**
pLC1031	*HygB-TAR-tetO*	[6]
pLC1081	pV1093	[7]
pLC1100	pFA-3HA-ARG	[8]

Supplementary Table 3: Oligonucleotides used in this study.

Accession Number	Name	Sequence (5'-3')
oLC2637	CaROB1AB +1346F	TGCTGCTTTGATAGCTTCTGG
oLC2888	CaROB1-372F	CCTCATGAATACTCAGCTGAG
oLC4714	tetOp+488F	TCGTTTCTGATGGGCTTTTC
oLC5570	CdARG4+1309-F	AGTGTGGAAAGAAGAGATGC
oLC6393	CaYAK1AB-91-F	AACGCAAGAGATCACATACC
oLC6926	SNR52/F	AAGAAAGAAAGAAAACCAGGAGTGAA
oLC6927	sgRNA/R	ACAAATATTTAAACTCGGGACCTGG
oLC6928	SNR52/N	GCGGCCGCAAGTGATTAGACT
oLC6929	sgRNA/N	GCAGCTCAGTGATTAAGAGTAAAGATGG
oLC8303	ROB1+2391R	CATCCCTTCTTCTTTGTTCC
oLC8730	CaOCA6 -3 F	ACAATGTCACAAATTGTCCC
oLC8740	CaOCA6 -262 F	TAGTTGTTGGTGTGCCTACC
oLC8741	CaOCA6 +475 R	TGTATTTCGCCGTGGAATCC
oLC8759	CaROB1 + 3521 R	CTCAAAGTGGGTGCTATAGG
oLC8906	ROB1 + 3353 + ARG4 up homology R	TCGATACATTTGCGGTACAGAAATGTTCTT AATCTTCGGGTTTGAGTACC
oLC8907	ARG4 -1033 + ROB1 ds homology F	CGGGTACTCAAACCCGAAGATTAAGAACA TTTCTGTACCGCA
oLC8908	ARG4 + 3211 + ROB1 ds homology R	ACTACTACTACTACACTCTAACACGGATA AATACACCCTTTAGTCGGTGGTGGAAATG CACGTTTGATTTACAAAAGCTATTTGCATC GT
oLC8916	OCA6 + 917 + ARG4 up homology R	TCGATACATTTGCGGTACAGAAATGTTCTT AAGAATTGGACATGACTCCG
oLC8917	ARG4 -1033 + OCA6 ds homology F	CCGGCCAATACGGAGTCATGTCCAATTCTT AAGAACATTTCTGTACCGCA

oLC8918	ARG4 + 3211 + OCA6 ds homology R	AAGCGGTTCAAAATGACCGCCAGTGGTGGATTTCACAAATTGGAAACTTTTGTGACTTACCCTGTGAATGACAAAAGCTATTTGCATCGT
oLC9199	oLC9199_SNR52/R_Yak1tag	CCACATTGTTATTCTTATTCCAAATTAAAAATAGTTTACGCAAGTC
oLC9200	oLC9200_SNR52/R_Yak1tag	GAATAAGAATAACAATGTGGGTTTTAGAGCTAGAAATAGCAAGTTAAA
oLC9201	oLC9201_Yak1 del F	TCACATACCATTAATAATATATAAGACCAACCATTGTAACCACACAAAGTATCACAGTATCACCGACAAATTTATACATAATGGCGGTCGACGGATCCCC
oLC9203	oLC9203_Yak1 del/tag R	AAACAAGATTGTTACATAAACAAAATTAATTAAAAAGTATCATTAAAGAGTATAAAACTTAATACTGGTCAACCTCCCCCTCTCGATGAATTCGAGCTCG
oLC9204	oLC9204_Yak1 orf check F	CAAATTCCTGGATTCCGTAACCCTTGG
oLC9205	oLC9205_Yak1 orf check R	GCAATTCCAGCAGGACCAGAAGG
oLC9206	oLC9206_Yak1 tag check F	CAATGATTGAAAAAGAATATCATGATCGA
oLC9207	oLC9207_Yak1 tag check R	TATAAACCATATTCAGTTCTTAACAA
oLC9452	CaYAK1_gRNA F	GATACTTTGTGTGGTTACAAGTTTTAGAGCTAGAAATAGCAAGTTAAA
oLC9453	CaYAK1_gRNA R	TTGTAACCACACAAAGTATCCAAATTAAAAATAGTTTACGCAAGTC
oLC9512	oLC9512_1031_tetO-Yak1 F	AATTCTGATCCTCCCCCTTTTTCCCTCAAGTTCAAACAAAACGCAAGAGATCACATACCATTAATAATATATAAGACCAAGACGTCGTATAGTGCTTGCT
oLC9513	oLC9513_1031_tetO-Yak1 R	TGCCAATTTCCTCCTATAGAATTGTGTCGATTAAAATTATAATTATAATTAGAACTGTTGTTATTGTTATTATATGCCATCGACTATTTATATTTGTATG

SUPPLEMENTARY REFERENCES

1. Abbey, D. A. *et al.* YMAP: A pipeline for visualization of copy number variation and loss of heterozygosity in eukaryotic pathogens. *Genome Med.* **6**, 1–15 (2014).
2. Noble, S. M. & Johnson, A. D. Strains and strategies for large-scale gene deletion studies of the diploid human fungal pathogen *Candida albicans*. *Eukaryot. Cell* **4**, 298–309 (2005).
3. Staab, J. F., Bahn, Y. S.& Sundstrom, P. Integrative, multifunctional plasmids for hypha-specific or constitutive expression of green fluorescent protein in *Candida albicans*. *Microbiology* **149**, 2977–2986 (2003).
4. Roemer, T. *et al.* Large-scale essential gene identification in *Candida albicans* and applications to antifungal drug discovery. *Mol. Microbiol.* **50**, 167–181 (2003).
5. Polvi, E. J. *et al.* Functional divergence of a global regulatory complex governing fungal filamentation. *PLOS Genet.* **15**, e1007901 (2019).
6. Kim, S. H. *et al.* Genetic analysis of *Candida auris* implicates Hsp90 in morphogenesis and azole tolerance and Cdr1 in azole resistance. *mBio* **10**, e02529-18 (2019).
7. Vyas, V. K., Barrasa, M. I. & Fink, G. R. A *Candida albicans* CRISPR system permits genetic engineering of essential genes and gene families. *Sci. Adv.* **1**, e1500248 (2015).
8. Zhang, A. *et al.* The Tlo proteins are stoichiometric components of *Candida albicans* mediator anchored via the Med3 subunit. *Eukaryot. Cell* **11**, 874–884 (2012).
