# Supplementary material for: A small molecule produced by Lactobacillus species blocks Candida albicans filamentation by inhibiting a DYRK1-family kinase
Source: Nat Commun. 2021 Oct 22;12:6151. doi: 10.1038/s41467-021-26390-w (PMC8536679; doi:10.1038/s41467-021-26390-w)
Supplement: Supplementary file 2 — Reporting Summary [file 41467_2021_26390_MOESM2_ESM.pdf]

nature research

Corresponding author(s): Jessie MacAlpine, Martin Daniel-Ivad, Zhongle Liu, Junko Yano, Nicole M. Revie, Robert T. Todd, Peter J. Stogios, Hiram Sanchez, Teresa O'Meara, Thomas A. Tompkins, Alexei Savchenko, Anna Selmecki, Amanda O. Veri, David R. Andes, Paul L. Fidel Jr., Nicole Robbins, Justin Nodwell, Luke Whitesell, and Leah E. Cowen

Last updated by author(s): Sep 21, 2021

Reporting Summary

Nature Research wishes to improve the reproducibility of the work that we publish. This form provides structure for consistency and transparency in reporting. For further information on Nature Research policies, see our Editorial Policies and the Editorial Policy Checklist.

Statistics

For all statistical analyses, confirm that the following items are present in the figure legend, table legend, main text, or Methods section.

n/a	Confirmed	
☐	☒	The exact sample size (*n*) for each experimental group/condition, given as a discrete number and unit of measurement
☐	☒	A statement on whether measurements were taken from distinct samples or whether the same sample was measured repeatedly
☐	☒	The statistical test(s) used AND whether they are one- or two-sided<br>*Only common tests should be described solely by name; describe more complex techniques in the Methods section.*
☒	☐	A description of all covariates tested
☒	☐	A description of any assumptions or corrections, such as tests of normality and adjustment for multiple comparisons
☐	☒	A full description of the statistical parameters including central tendency (e.g. means) or other basic estimates (e.g. regression coefficient) AND variation (e.g. standard deviation) or associated estimates of uncertainty (e.g. confidence intervals)
☐	☒	For null hypothesis testing, the test statistic (e.g. *F*, *t*, *r*) with confidence intervals, effect sizes, degrees of freedom and *P* value noted<br>*Give P values as exact values whenever suitable.*
☒	☐	For Bayesian analysis, information on the choice of priors and Markov chain Monte Carlo settings
☒	☐	For hierarchical and complex designs, identification of the appropriate level for tests and full reporting of outcomes
☒	☐	Estimates of effect sizes (e.g. Cohen's *d*, Pearson's *r*), indicating how they were calculated

Our web collection on statistics for biologists contains articles on many of the points above.

Software and code

Policy information about availability of computer code

Data collection	CytExpert (v2.4)was used for flow cytometry data collection
Data analysis	YMAP (v1.0), Phyre2 (v2.0), YASARA (v20,12,24), Autodock Vina (v1.1.2), PyMOL (v2.5.0), Prism 6, Bio-Rad CFX Manager (v3.1), MuTect (v1.1.4.)

For manuscripts utilizing custom algorithms or software that are central to the research but not yet described in published literature, software must be made available to editors and reviewers. We strongly encourage code deposition in a community repository (e.g. GitHub). See the Nature Research guidelines for submitting code & software for further information.

Data

Policy information about availability of data

All manuscripts must include a data availability statement. This statement should provide the following information, where applicable:

- Accession codes, unique identifiers, or web links for publicly available datasets
- A list of figures that have associated raw data
- A description of any restrictions on data availability

Source data are provided with this paper. All additional data, including raw data and images associated with all figures, is available from the corresponding author, L.E.C, upon reasonable request.

Field-specific reporting

Please select the one below that is the best fit for your research. If you are not sure, read the appropriate sections before making your selection.

☒ Life sciences ☐ Behavioural & social sciences ☐ Ecological, evolutionary & environmental sciences

For a reference copy of the document with all sections, see nature.com/documents/nr-reporting-summary-flat.pdf

Life sciences study design

All studies must disclose on these points even when the disclosure is negative.

Sample size	In order to use statistical analysis, sample size was always n=3 or greater. Murine explant experiments used 3-5 mice per group based on historical variability data within groups. All experiments were performed in biological duplicate or greater with little deviation between replicates.
Data exclusions	In flow cytometry experiments, events were excluded from calculation of median fluorescence intensity (MFI) by gating on forward and side scatter parameters to eliminate debris and multi-cell clumps that would skew data. Gating removed less than 15% of all acquired events. No other data were excluded from analysis.
Replication	All attempts at replication were successful. Unless otherwise noted, all experiments are representative of at least two biological replicates.
Randomization	For all animal studies, animals were randomly assigned to experimental groups.
Blinding	Blinding was not relevant to this study as this was not an observational study with no opportunity for bias to factor into quantitative results. All assays had a quantitative output, rather than qualitative, and therefore, blinding was not required to eliminate user bias.

Reporting for specific materials, systems and methods

We require information from authors about some types of materials, experimental systems and methods used in many studies. Here, indicate whether each material, system or method listed is relevant to your study. If you are not sure if a list item applies to your research, read the appropriate section before selecting a response.

Materials & experimental systems

n/a	Involved in the study	
☒	☐	Antibodies
☐	☒	Eukaryotic cell lines
☒	☐	Palaeontology and archaeology
☐	☒	Animals and other organisms
☒	☐	Human research participants
☒	☐	Clinical data
☒	☐	Dual use research of concern

Methods

n/a	Involved in the study	
☒	☐	ChIP-seq
☐	☒	Flow cytometry
☒	☐	MRI-based neuroimaging

Eukaryotic cell lines

Policy information about cell lines

Cell line source(s)	HepG2 cells were provided by ATCC. (ATCC HB-8065).
Authentication	Cell line was not authenticated as a specific tissue of origin was non-critical to the validity of the results reported.
Mycoplasma contamination	All cell lines tested negative for Mycoplasma contamination.
Commonly misidentified lines (See ICLAC register)	No commonly misidentified cell lines were used.

Animals and other organisms

Policy information about studies involving animals; ARRIVE guidelines recommended for reporting animal research

Laboratory animals	C3H/HeN (purchased from Charles River), female mice, 6-weeks old for mouse vaginal explants and Spraw Daley (purchased from Envigo), male rats, 10 weeks old for the rat catheter experiments.
Wild animals	This study did not involve wild animals.

Field-collected samples: The study did not involve samples collected from the field.

Ethics oversight: Murine mouse tissues for the vaginal explant were acquired and analyzed using protocols approved by the Institutional Animal Care and Use Committee (IACUC) of Louisiana State University Health Sciences Center (Protocol 3663). Rat catheter biofilm animal procedures were approved by the Institutional Animal Care and Use Committee at the University of Wisconsin-Madison according to the guidelines of the Animal Welfare Act, The Institute of Laboratory Animals Resources Guide for the Care and Use of Laboratory Animals, and Public Health Service Policy. The approved animal protocol number is DA0031.

Note that full information on the approval of the study protocol must also be provided in the manuscript.

Flow Cytometry

Plots

Confirm that:

☒ The axis labels state the marker and fluorochrome used (e.g. CD4-FITC).

☒ The axis scales are clearly visible. Include numbers along axes only for bottom left plot of group (a 'group' is an analysis of identical markers).

☒ All plots are contour plots with outliers or pseudocolor plots.

☒ A numerical value for number of cells or percentage (with statistics) is provided.

Methodology

Sample preparation: Cells used in the flow cytometry experiments include the HWP1p-GFP C. albicans strain. Supplementary Table 1 for this study includes more information on the strain. C. albicans cells were sub-cultured from a saturated overnight culture to an OD600 of 0.1 in YPD medium and grown for 6 hours at 42°C in the absence or presence of 1-ABC or Lactobacillus-conditioned medium. Cells were then pelleted, washed once with PBS, resuspended in PBS, and diluted 1:10 in 1 mL PBS. 250 µL of each sample were added to a flat-bottom transparent 96-well plate (Beckman Coulter).

Instrument: CytoFLEX S (Beckman Coulter).

Software: CytExpert Software (v2.4).

Cell population abundance: The cell population post-sort was approximately 80% of the population.

Gating strategy: The purpose of the gating strategy was to capture the C. albicans population undergoing the morphogenesis process. The gating strategy captured cells with a FSC-A between 40x10^4 and 280x10^4 and SSC-A between 20x10^4 and 190x10^4.

☒ Tick this box to confirm that a figure exemplifying the gating strategy is provided in the Supplementary Information.
